# Manipulating plant RNA-silencing pathways to improve the gene editing efficiency of CRISPR/Cas9 systems

**DOI:** 10.1186/s13059-018-1529-7

**Published:** 2018-09-28

**Authors:** Yanfei Mao, Xiaoxuan Yang, Yiting Zhou, Zhengjing Zhang, Jose Ramon Botella, Jian-Kang Zhu

**Affiliations:** 1 Shanghai Center for Plant Stress Biology, CAS Center of Excellence in Molecular Plant Sciences, Chinese Academy of Sciences, Shanghai, 200032 People’s Republic of China; 2 University of Chinese Academy of Sciences (CAS), Beijing, 100049 People’s Republic of China; 3 School of Agriculture and Food Sciences, University of Queensland, Brisbane, QLD 4072 Australia; 4 Department of Horticulture and Landscape Architecture, Purdue University, West Lafayette, IN 47907 USA

**Keywords:** CRISPR, Cas9, Genome editing, Viral suppressor, RNA silencing, TBSV p19

## Abstract

**Background:**

The CRISPR/Cas9 system, composed of a single-guide RNA for target recognition and a Cas9 protein for DNA cleavage, has the potential to revolutionize agriculture as well as medicine. Even though extensive work has been done to improve the gene editing activity of CRISPR/Cas9, little is known about the regulation of this bacterial system in eukaryotic host cells, especially at the post-transcriptional level.

**Results:**

Here, we evaluate the expression levels of the two CRISPR/Cas9 components and the gene editing efficiency in a set of Arabidopsis mutants involved in RNA silencing. We find that mutants defective in the post-transcriptional gene-silencing pathway display significantly higher Cas9 and sgRNA transcript levels, resulting in higher mutagenesis frequencies than wild-type controls. Accordingly, silencing of AGO1 by introduction of an AGO1-RNAi cassette into the CRISPR/Cas9 vector provides an increase in gene editing efficiency. Co-expression of the viral suppressor p19 from the tomato bushy stunt virus to suppress the plant RNA-silencing pathway shows a strong correlation between the severity of the phenotypic effects caused by p19 and the gene editing efficiency of the CRISPR/Cas9 system for two different target genes, AP1 and TT4.

**Conclusions:**

This system has useful practical applications in facilitating the detection of CRISPR/Cas9-induced mutations in T1 plants as well as the identification of transgene-free T2 plants by simple visual observation of the symptom severity caused by p19. Our study shows that CRISPR/Cas9 gene editing efficiency can be improved by reducing RNA silencing in plants.

**Electronic supplementary material:**

The online version of this article (10.1186/s13059-018-1529-7) contains supplementary material, which is available to authorized users.

## Background

The recent development of the CRISPSR/Cas9 system has revolutionized genetic research, and its practical applications are emerging in multiple fields, from agriculture to human health. The best known CRISPR/Cas9 system is derived from the *Streptococcus pyogenes* adaptive immune system where it is used to silence invasive DNA molecules [1]. This RNA-directed gene editing system is composed of a Cas9 nuclease and two bound RNAs, which form a partially paired duplex after processing of their primary transcripts by bacterial RNase III [2]. For practical applications, the RNA duplex has been shortened and linked together to create a single-guide RNA (sgRNA) with a stem-loop structure at the fusion site, resulting in higher gene editing efficiencies in vivo and in vitro compared to the natural version [3, 4]. A number of vectors have been developed to deliver the simplified CRISPR/Cas9 system into host cells, most of which use strong constitutive promoters to drive a high expression of both components, sgRNA and Cas9 [4–6]. Although many of these vectors can induce efficient targeted gene editing in plants, high transgene expression levels carry the risk of activating “transgene-silencing” mechanisms in the host cells.

The evolutionarily conserved transgene silencing process is a manifestation of the RNA-silencing pathway [7]. This pathway is used as defense against viral and sub-viral pathogens, which can produce dsRNAs as replication intermediates or single-stranded RNAs with secondary structures [8]. In addition to viral transcripts, dsRNAs can also be formed by the annealing of overlapping complementary transcripts or from hairpin precursors [7]. At the initial stage of RNA-silencing, dsRNAs are processed into 21–24-nt small interfering RNAs (siRNAs), by the DICER RNase III enzyme [9]. *Arabidopsis thaliana* encodes four DICER-like (DCL) proteins (DCL1-4) [10]. DCL1 primarily functions in the biosynthesis of microRNAs to silence endogenous messenger RNAs while DCL3 contributes to the defense against viruses by producing 24-nt viral-derived siRNAs [11, 12]. DCL2 and DCL4 have partially redundant roles in RNA silencing, producing 21–22-nt small RNAs upon viral attack although DCL4 seems to contribute more than DCL2 in the defense hierarchy [13].

The siRNA duplexes are incorporated into RNA-induced silencing complexes (RISCs) to direct the silencing of both coding and non-coding RNAs by sequence complementarity [14–16]. ARGONAUTE proteins are core components of the RISC complex, many of which have partially overlapping roles in antiviral defense and in the endogenous silencing pathways [17, 18]. For example, the AGO1 and AGO2 proteins can bind endogenous miRNAs as well as virus-derived 21–22-nt siRNAs in Arabidopsis and tobacco [19, 20]. AGO4 has a crucial role in the RNA-directed DNA methylation pathway and participates in the transcriptional suppression of some viral chromatin [21, 22].

While plants developed RNA-silencing pathways to fight viral infections, viruses also evolved strategies to counteract this defense mechanism [23]. The primary counter strategy involves the production of silencing suppressor proteins by RNA and DNA viruses [24]. Viral suppressors of RNA silencing (VSRs) have been extensively studied, and even though they all aim to suppress the plant RNA-silencing machinery, they can use very different strategies including inhibition of DICER and RISC activities, dsRNA/siRNA-sequestration, and destabilization of AGO proteins [24]. One of the best characterized VSRs is the p19 protein from the tomato bushy stunt virus (TBSV) [25, 26]. This protein has been widely used to enhance the transient expression of transgenes in many plant species [27–29], although its use in stable transformation has been limited by the developmental alterations caused by its ectopic expression [29–31].

With the broad adoption of the CRISPR/Cas9 system, many studies have focused at the transcriptional level in order to improve editing efficiency; however, little attention has been devoted to potential post-transcriptional regulation. In this work, we evaluated the gene editing activity of a previously characterized CRISPR/Cas9 system in a set of Arabidopsis mutants involved in different steps of the RNA-silencing pathway. Some of the tested mutants show higher mutagenesis frequency than wild type, coinciding with higher levels of sgRNA and Cas9 transcripts. A similar improvement in CRISPR/Cas9-mediated gene editing efficiency was obtained by silencing of *AGO1* using RNA interference in transgenic plants. Based on these observations, we tested the effect of expressing the viral suppressor p19 to counteract the plant RNA-silencing pathway in stably transformed Arabidopsis. A significant improvement in mutagenesis efficiency was observed in plants exhibiting strong p19-induced leaf developmental phenotypes together with a dramatic increase in the sgRNA and Cas9 transcript levels. As an additional benefit, the abnormal leaf phenotype induced by p19 expression or *AGO1* silencing can be used as a visual indicator to facilitate the screening of gene-edited plants in the T1 generation. We propose that the existing CRISPR/Cas9 systems can be optimized by reducing RNA silencing in plants.

## Results

### Analysis of CRISPR/Cas9-targeted gene editing efficiency in RNA-silencing pathway mutants

**Fig. 1:**
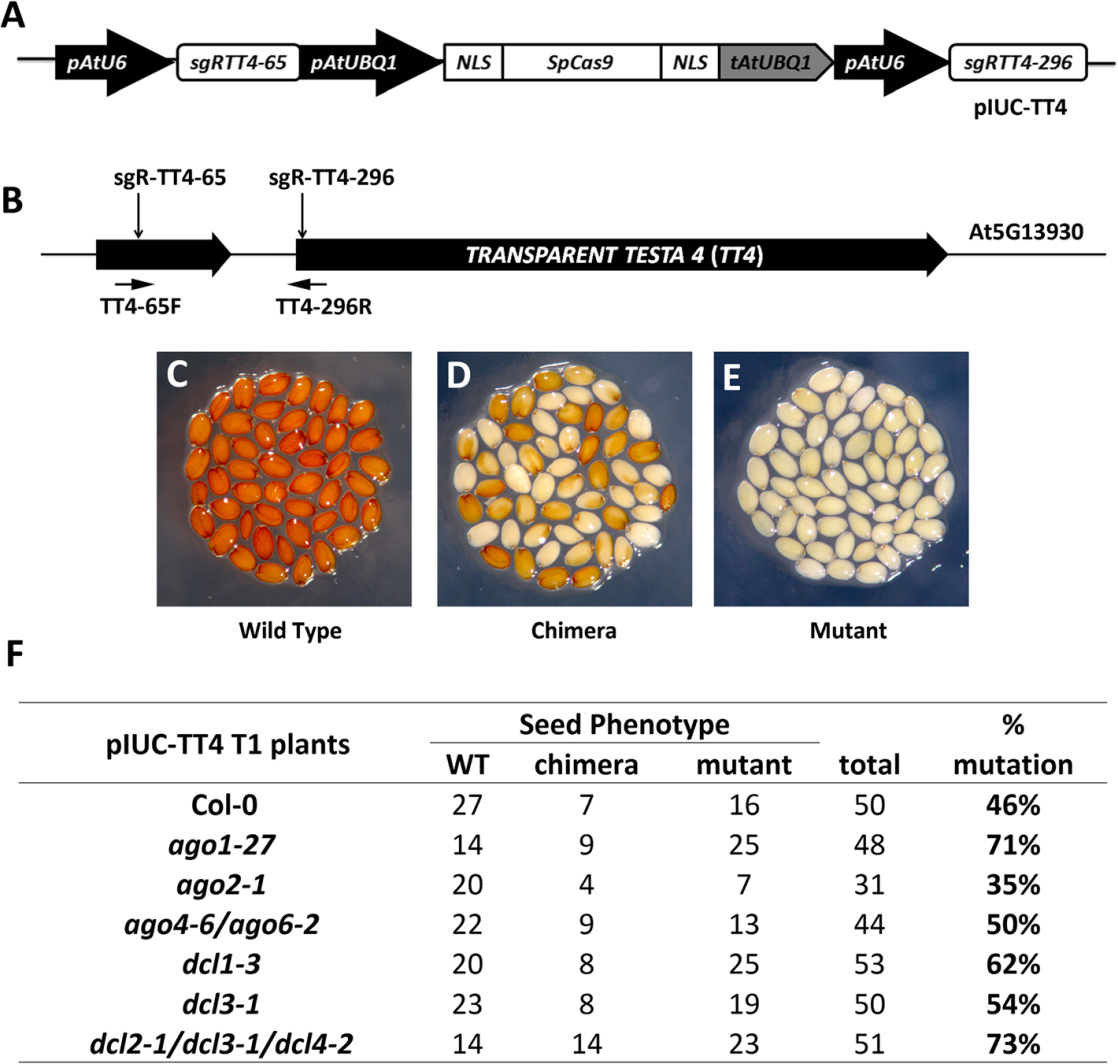
Analysis of CRISPR/Cas9-induced targeted gene mutations in Arabidopsis RNA-silencing pathway mutants. **a** An ectopically expressed CRISPR/Cas9 system (pIUC-TT4) designed for targeted gene mutagenesis in Arabidopsis. *pAtU6*, Arabidopsis U6-26 promoter; sgRNA, single guide RNA; *pAtUBQ1*, Arabidopsis *UBIQUITIN 1* promoter; *NLS*, SV40 nuclear localization signal; *SpCas9*, *Streptococcus pyogenes* Cas9 gene; *tAtUBQ1*, Arabidopsis *UBIQUITIN 1* terminator. **b** Schematic diagram of the *TT4* gene. The vertical arrows indicate the positions of the sgRNA target sites. The horizontal arrows indicate the position of primers used for analyzing the relative integrity (RI) of *TT4* gene. **c**–**e** T1 seeds obtained from pIUC-TT4 transgenic lines exhibited different severities of seed coat phenotypes. **c** Wild-type seeds. **d** Chimeric seeds. **e** Mutated seeds. **f** Phenotype analysis of CRISRP/Cas9-induced mutations in different silencing pathway mutants. % of mutation = (chimera + mutant)/total

Plant CRISPR/Cas9 expression cassettes usually contain strong constitutive promoters to drive sgRNA and Cas9 expression at high levels in order to achieve efficient gene editing [32]. High transgene expression may trigger the cellular-silencing machinery, which may limit the efficiency of CRISPR-induced gene editing. To test this hypothesis, we used a previously reported CRISPR/Cas9 vector (Fig. 1a) to transform a set of Arabidopsis mutants involved in RNA silencing, including *ago1-27*, *ago2-1*, *ago4-6/ago6-2*, *dcl1-3*, and *dcl2-1/dcl3-1/dcl4-2*. The *TRANSPARENT TESTA 4* (*TT4*) gene was selected as the CRISPR target (Fig. 1b). Mutations in this gene stop the production of brown pigment in Arabidopsis seeds providing an easily observable phenotype to determine the extent of gene editing in T1 and T2 plants. Three different types of visual phenotypes were expected in the seeds from the T1 population. “Wild-type” plants should produce completely normal brown seeds while “mutant” plants should produce entirely pale yellow seeds (Fig. 1c, e). In contrast, “chimera” plants should produce seeds with a mixed phenotype showing pale and dark sectors (Fig. 1d).

The mutation frequencies in the *TT4* gene in each mutant background were calculated by adding up the number of “mutant” and “chimera” plants (Fig. 1f). Compared to Col-0 plants (46%), mutagenesis frequency was increased to 71% in *ago1-27*, 62% in *dcl1-3*, and 73% in*dcl2-1/dcl3-1/dcl4-2* triple mutants. However, slight changes were observed in *ago4-6/ago6-2* double mutants (50%) and *dcl3-1* (54%), and a decrease was evident in *ago2-1* mutants (35%).

**Fig. 2:**
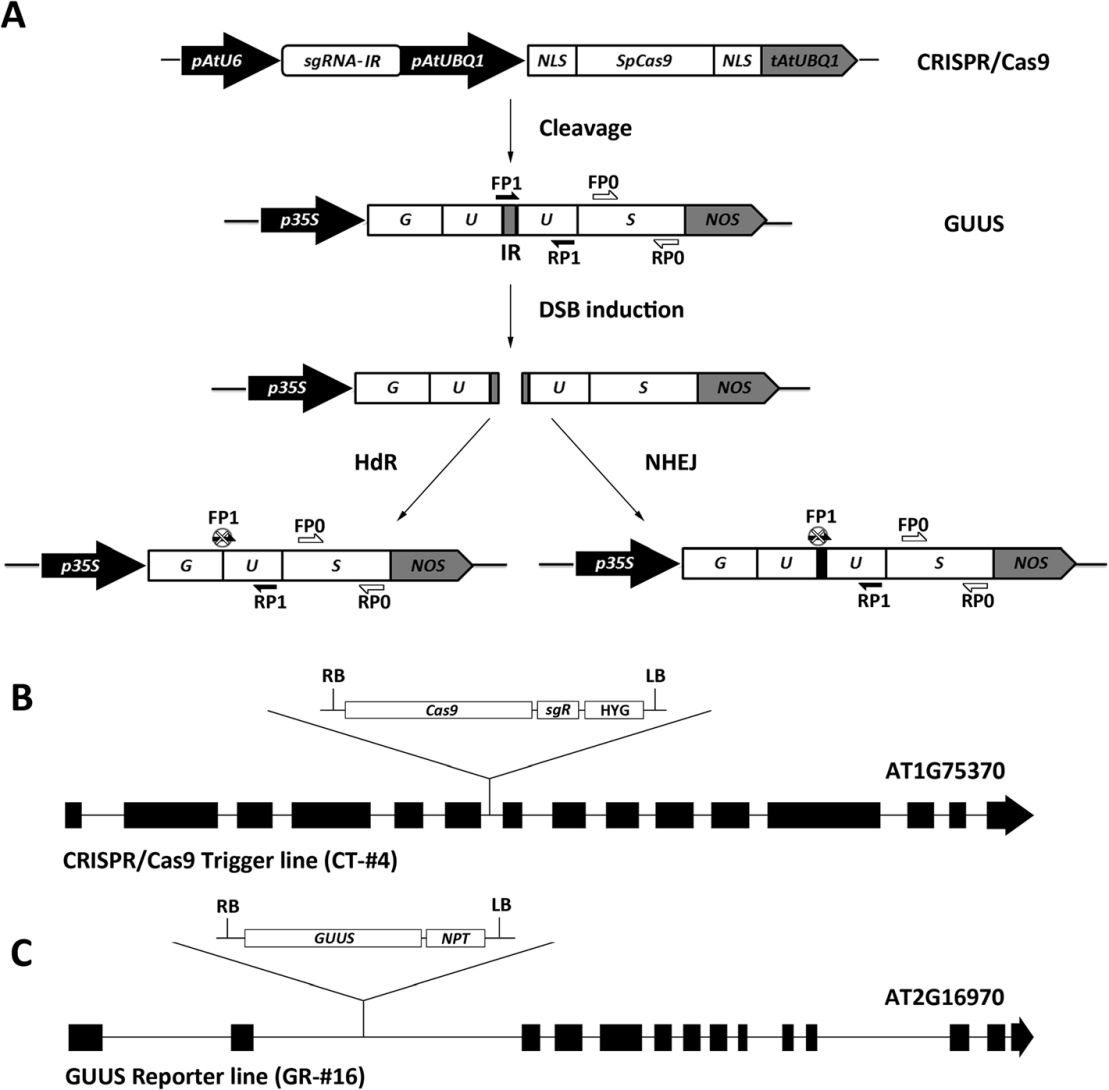
Design of a GUUS reporter system to evaluate the gene editing activity of CRISPR/Cas9 in Arabidopsis transgenic plants. **a** Schematics of the GUUS reporter construct and the CRISPR/Cas9 construct used for plant transformation. CRISPR/Cas9 induces cleavage of the GUUS reporter gene at the intergenic region (IR). The double-stranded break (DSB) could be subsequently repaired by the homology-dependent recombination pathway (HdR) or the error-prone non-homologous end joining pathway (NHEJ). In both cases, the sgRNA binding site in the IR would be mutated hampering binding of the FP1 primer. In this way, the PCR amplification efficiency of the “U” fragment would be reduced compared to the non-targeted “S” fragment. FP1/RP1, PCR primers designed to amplify the “U” fragment of the GUUS gene. FP0/RP0, PCR primers designed to amplify the “S” fragment of GUUS gene. **b** A single copy CRISPR/Cas9 transgenic line was identified with the T-DNA inserted into the sixth intron of the At1g75730 gene. Cas9, the Cas9 expression module as shown in **a**; sgR, sgRNA expression module as shown in **a**; HYG, hygromycin resistance module derived from pCAMBIA1300; LB, T-DNA left border; RB, T-DNA right border. **c** A single copy GUUS reporter transgenic line was identified with the T-DNA inserted into the second intron of the At2g16970 gene. GUUS, GUUS expression module as shown in **a**; NPT, Neomycin resistance module derived from pCAMBIA2300

To validate the effect of different RNA-silencing pathway components on the activity of the CRISPR/Cas9 system, we used a previously constructed CRISPR/Cas9 expression vector targeting an intergenic region (IR) between a partially duplicated GUUS reporter gene (Fig. 2a). CRISPR/Cas9-mediated double strand breaks in the IR can be repaired either via the homology-dependent recombination (HdR) pathway to restore a functional GUS gene or via the error-prone non-homologous end joining (NHEJ) pathway that can introduce mutations at the target site. A PCR primer (FP1) designed for the target site will have its PCR amplification efficiency reduced by the presence of NHEJ-induced mutations, or completely eliminated in the case of HdR-mediated recombination when used in combination with a reverse PCR primer in the U fragment (Fig. 2a, Additional file 1: Figure S1) [33]. A second pair of primers was designed for the non-targeted “S” fragment that should not be affected by CRISPR/Cas9-induced modifications. In this way, the CRISPR/Cas9 gene editing efficiency can be estimated by comparing the GUUS gene relative integrity (RI) as a ratio between the amplification efficiency of the U fragment versus the S fragment (RI = U/S) in different samples [34] .

**Fig. 3:**
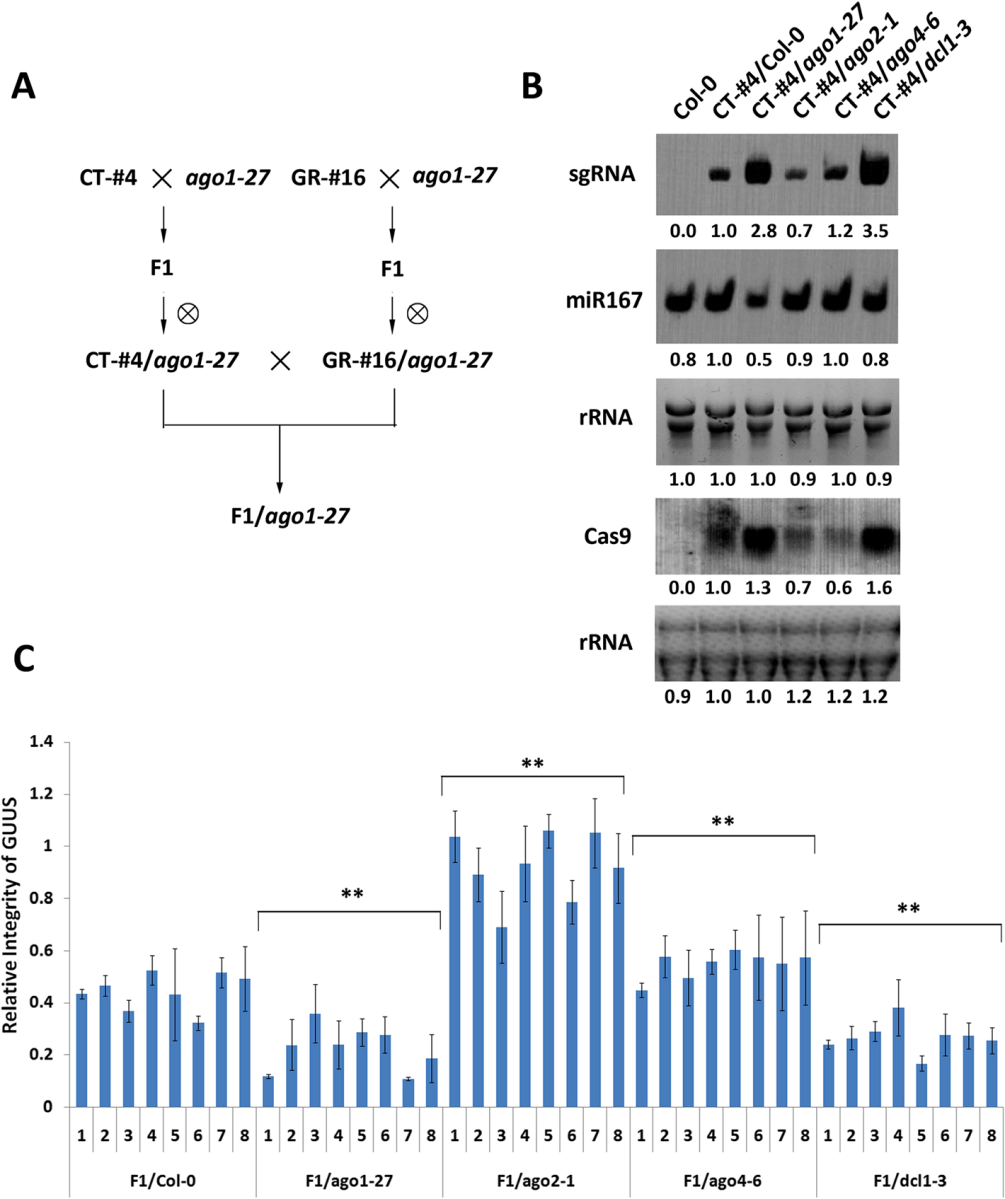
Gene expression levels and editing activity of CRISPR/Cas9 in RNA-silencing pathway mutants. **a** A pipeline used to generate F1 hybrids of CRISPR/Cas9 and GUUS lines in different mutant backgrounds, *ago1-27* is shown as an example. **b** Northern blot showing sgRNA, miR167, and Cas9 transcript levels in RNA-silencing pathway mutants. **c** Relative integrity (RI) of the GUUS reporter gene in different samples was detected by real-time PCR. Each column indicates means ± SD of three technical replicates. For each mutant background, eight individuals were analyzed as biological replicates. **Indicates significant difference at *p* < 0.01 under two-tailed Student’s *t* test

To enable comparative analysis of targeted gene editing efficiency in different mutants, the CRISPR/Cas9 construct and the GUUS reporter construct were first introduced separately into Arabidopsis. Several T2 lines showing a Mendelian 3:1 segregation ratio were selected and their T-DNA insertion sites determined to ensure that no RNA-silencing pathway genes had been inadvertently affected. Two homozygous transgenic T3 lines were finally selected for further work, one carrying the CRISPR/Cas9 construct (CT-#4) and the other carrying the GUUS reporter construct (GR-#16) (Fig. 2b, c). These two lines were then crossed with different post-transcriptional gene-silencing (PTGS) pathway mutants respectively to obtain homozygous CT-#4 and GR-#16 lines in mutant backgrounds in the F2 generation. As an example, the *ago1-27* mutant was crossed with homozygous CT-#4 and GR-#16 lines to generate homozygous CT-#4/*ago1-27* and GR-#16/*ago1-27* individuals in F2 generation. For mutagenesis analysis, the homozygous CT-#4/*ago1-27* and GR-#16/*ago1-27* individuals were cross-pollinated to produce the heterozygous CT-#4 and GR-#16 transgenes in homozygous *ago1-27* mutant plants (Fig. 3a). Although time consuming, this strategy was essential to discard effects caused by integration of the T-DNAs in different genomic loci for each mutant background.

RI values for the GUUS reporter gene were determined in the *ago1-27*, *ago2-1*, *ago4-6*, and *dcl1-3* mutant backgrounds as well as in a wild-type Col-0 line as control. Due to the complexity of the approach, we did not use it for the *ago4-6/ago6-2* double and *dcl2-1/dcl3-1/dcl4-2* triple mutants (analyzed in Fig. 1f). Compared to the Col-0 background, the RI values for the GUUS reporter gene were significantly reduced in the *ago1-27* and *dcl1-3* mutant backgrounds (Fig. 3c), reflecting higher mutagenesis efficiencies in agreement with our above results using the TT4 gene (Fig. 1f). The *ago2-1* mutant produced the highest RI values, also consistent with the low mutation efficiency observed for the TT4 gene (Figs. 3c and 1f). Finally, while the *ago4-6/ago6-2* mutants showed similar mutagenesis efficiency to Col-0 in the TT4 assays, the single *ago4-6* showed increased GUUS RI values, indicating reduced mutation efficiency.

Quantification of the transcript levels for both elements of the CRISPR/Cas9 system, sgRNA and Cas9, revealed a strong correlation between expression levels and mutation efficiency (Fig. 3b, c). The CT-#4/*ago1-27* and CT-#4/*dcl1-3* transgenic lines showed elevated levels of Cas9 and sgRNA compared to the CT-#4/Col-0 control, while CT-#4/*ago2-1* displayed lower Cas9 and sgRNA levels than CT-#4/Col-0 (Fig. 3b). Interestingly, CT-#4/*ago4-6* showed a reduction in Cas9 levels but not in sgRNA levels, possibly indicating that the AGO4-mediated RNA-silencing pathway does not affect the relatively small sgRNA transcript.

### Interference with the PTGS pathway leads to improvements in CRISPR/Cas9 gene editing efficiency in Arabidopsis

**Fig. 4:**
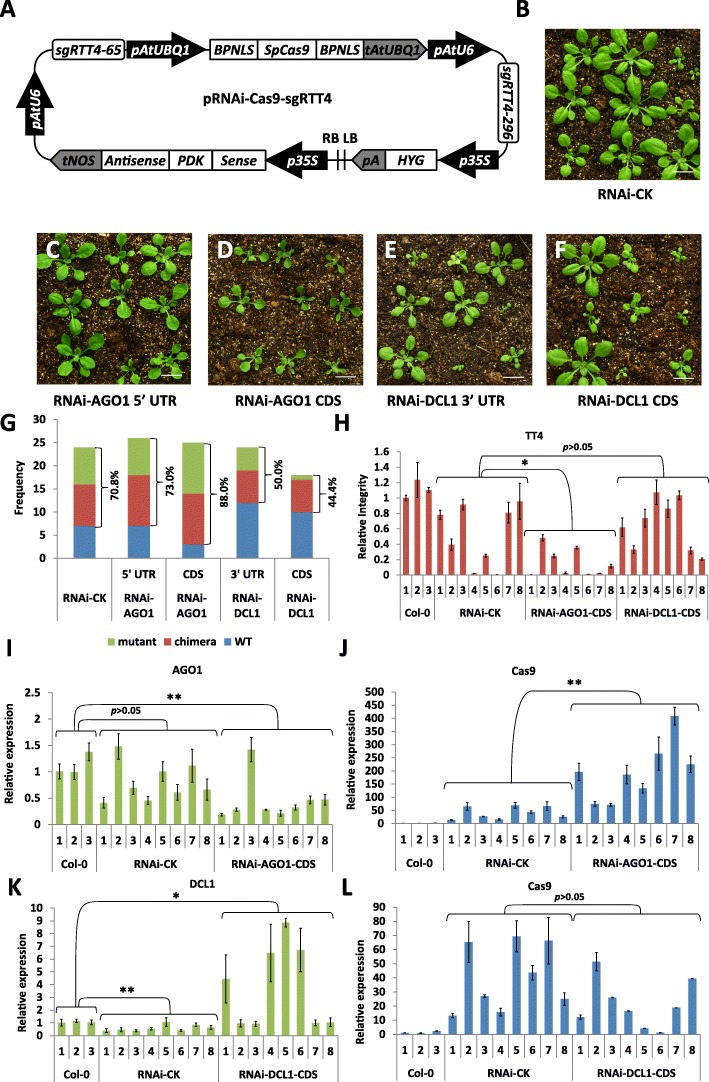
Improving CRISPR/Cas9 gene editing efficiency by interference with RNA-silencing pathway gene expression. **a** A schematic diagram of the CRISPR/Cas9 system containing an RNA interference construct. *BPNLS*, bipartite nuclear localization signal; Sense, sense fragment of targeted gene; PDK, PDK intron from pHellsgate; Antisense, antisense fragment of targeted gene. **b**–**f** T1 transgenic plants transformed with pRNAi-Cas9-sgRTT4 containing an empty RNA interference construct (RNAi-CK) (**b**), an AGO1 5′UTR RNAi construct (**c**), an AGO1 CDS RNAi construct (**d**), a DCL1 3′UTR RNAi construct (**e**) and a DCL1 CDS RNAi construct (**f**). Bar = 1 cm. **g** Histogram showing the frequency of WT, chimeras, and mutants in T1 populations transformed with different pRNAi-Cas9-sgRTT4 constructs. The numbers indicates % of mutation = (chimera + mutant)/total (**h**). Relative integrity of the *TT4* gene in RNAi-CK, RNAi-AGO1 CDS and RNAi-DCL1 CDST1 plants. Eight individual T1 plants were analyzed for each construct by real-time PCR. **i**, **j** Relative expression of *AGO1* (**i**) and *Cas9* (**j**) in Col-0, RNAi-CK, and RNAi-AGO1 CDS T1 plants determined by quantitative RT-PCR. **k**, **l** Relative expression of *DCL1* (**k**) and *Cas9* (**l**) in Col-0, RNAi-CK, and RNAi-DCL1 CDS T1 plants determined by quantitative RT-PCR. Each column in (**h**–**l**) indicates means ± SD of three technical replicates. The values of Col-0 samples were arbitrarily designated as 1. *Significant difference at *p* < 0.05 under two-tailed Student’s *t*-test. **Significant difference at *p* < 0.01 under two-tailed Student’s *t* test. “*p* > 0.05” indicates no significant difference

Given that *ago1* and *dcl1* mutants show increased gene editing efficiency, we designed a strategy to couple CRISPR/Cas9 with silencing of the *AGO1* and *DCL1* genes. For this purpose, we introduced additional elements into a nuclear localization signal optimized pIUC-TT4 CRISPR/Cas9 vector to trigger the RNA interference (RNAi) response in plant cells by producing hairpin RNA molecules (Fig. 4a, Additional file 1: Note S). Since the silencing efficiency of RNAi is dependent on the sequences of hairpin RNAs, we produced two CRISPR/RNAi constructs for *AGO1* using fragments from the 5′ untranslated region (UTR) and the coding region (CDS), while for *DCL1* we used the coding region and the 3’UTR. Characteristic developmental defects were observed in the T1 transgenic population [35] with lines containing the CDS *AGO1* RNAi construct showing the highest phenotype frequency and severity (Fig. 4b–d). To evaluate the CRISPR/Cas9-mediated gene editing efficiency in the RNAi populations, we calculated the mutagenesis frequency in the *TT4* gene by adding up the number of “mutant” and “chimera” plants. Compared to the control containing an empty RNAi cassette (RNAi-CK), mutagenesis frequency was increased in the RNAi-AGO1-CDS lines (88.0% vs 70.8%), while no substantial difference was observed for the RNAi-AGO1-5′UTR lines (73.0% vs 70.8%). Surprisingly, lines harboring both DCL RNAi constructs showed reduced frequency of CRISPR/Cas9-induced mutations (50.0% vs 70.8%, 44.4% vs 70.8%) (Fig. 4g).

To validate these results, we quantified Cas9 transcript levels and targeted gene mutation frequency in eight individuals randomly selected from the RNAi-AGO1-CDS and RNAi-DCL1-CDS populations. Analyses of the RNAi-AGO1-CDS samples showed efficient silencing of the *AGO1* gene in most tested seedlings (Fig. 4i), which coincided with a significant increase in Cas9 transcript levels compared to control samples (Fig. 4j). The relative integrity (RI) of the *TT4* gene was calculated as a ratio between the amplification efficiency of the targeted *TT4* region versus the non-targeted *ACTIN* region using primer pairs shown in Fig. 1b and Additional file 1: Table S1. The overall RI value of *TT4* gene in RNAi-AGO1-CDS plants was significantly lower than controls, suggesting that successful RNAi-mediated silencing of *AGO1* can improve the gene editing efficiency of CRISPR/Cas9 (Fig. 4h). In contrast, instead of silencing, half of the tested RNAi-DCL1-CDS samples showed higher *DCL1* expression levels (line #1, #4, #5 and #6) (Fig. 4k), suggesting the existence of some kind of feedback regulation. Although there was no significant difference in Cas9 expression and overall TT4 RI value between the RNAi-DCL1-CDS samples and control samples, the four RNAi-DCL1-CDS lines with increased DCL1 expression exhibited relatively lower Cas9 levels compared to the other RNAi-DCL1-CDS lines (Fig. 4l, h). These results further supported our initial observations that manipulation of the AGO1-directed RNA-silencing pathway can lead to improved CRISPR/Cas9 efficiency.

### Expression of the viral p19-silencing suppressor increases CRISPR/Cas9-mediated gene editing efficiency in Arabidopsis transgenic plants

**Fig. 5:**
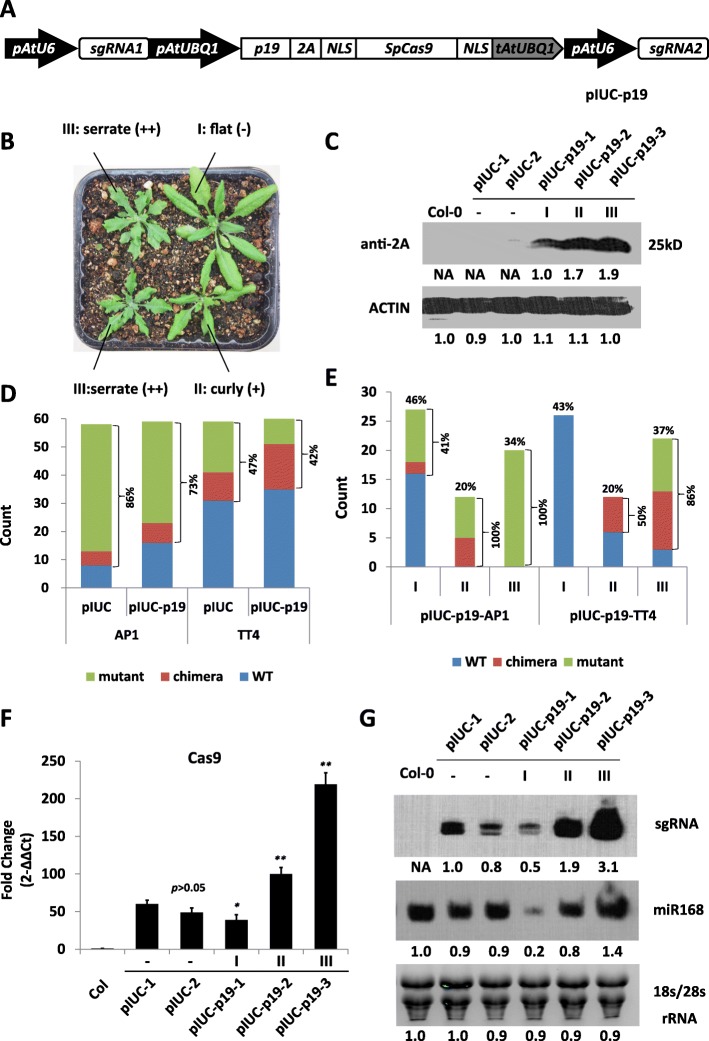
Co-expression of p19 and CRISPR/Cas9 in Arabidopsis transgenic plants. **a** Schematic diagram of the p19 co-expression CRISPR/Cas9 system, pIUC-p19. p19, tomato bushy stunt virus p19 gene; 2A, Porcine teschovirus-1 2A peptide. **b** T1 transformants exhibited leaf developmental phenotypes with different severities. “I”: WT looking plants; “II”: mild phenotype with curly leaves; “III”: severe phenotype with serrated leaves. **c** p19 protein levels in T1 transgenic lines exhibiting different leaf phenotype severities were detected by Western Blot using anti-2A antibodies. **d** Histogram showing the number of WT, chimeras and mutant plants in T1 populations transformed with pIUC and pIUC-p19. For each system, two different genes, *TT4* and *AP1*, were targeted. **e** Re-analysis of the data shown in (**d**) grouping plants according to the severity of their leaf phenotype. The numbers on the top of each column indicate the proportion of grouped phenotypes in the population **f** Cas9 transcript levels in T1 transgenic lines exhibiting different leaf phenotype severities determined by quantitative RT-PCR. The columns indicate mean ± SD of three biological replicates. Statistic was performed with *t* test in comparison to pIUC-1. *Significant difference at *p* < 0.05 under two-tailed Student’s *t* test. **Significant difference at *p* < 0.01 under two-tailed Student’s *t* test. “*p* > 0.05” indicates no significant difference. **g**, sgRNA and miR168 expression levels in T1 transgenic lines exhibiting different leaf phenotype severities detected by Northern blot analysis

As an alternative strategy to silencing *AGO1*, we studied the effect of a well-known viral silencing suppressor (VSR), the TBSV p19 protein, to counteract the effect of the AGO1-mediated RNA-silencing pathway. For this purpose, we constructed a vector allowing co-expression of p19 as part of the CRISPR/Cas9 expression cassette (Fig. 5a). CRISPR/Cas9 constructs targeting the *TT4*, and *APETALA1* (*AP1*) genes were made with or without the presence of p19. Strong ectopic expression of p19 in Arabidopsis results in a clear leaf developmental phenotype of serrated and curled leaves [30], making it easy to infer p19 expression levels in transgenic lines by simply observing leaf shapes. In our experiments, more than half of the T1 transgenic seedlings containing the p19 co-expression constructs exhibited the expected p19-induced leaf developmental phenotype. The T1 plants were classified into three types according to the severity of this developmental defect (Fig. 5b). Type I plants grew normal flat leaves, type II plants exhibited slightly curly leaves, and type III plants developed serrated and downward curved leaves (Fig. 5b). Western blot analysis confirmed a positive correlation between the severity of the leaf developmental phenotypes and the levels of p19 protein in the transgenic plants (Fig. 5c). To determine whether p19 ectopic expression improves CRISPR/Cas9 gene editing efficiency, the overall mutation frequencies in the two target genes, *AP1* and *TT4*, were calculated. Unexpectedly, a slight decrease in mutation frequency was observed in T1 populations transformed with p19-coexpressed CRISPR/Cas9 systems compared to those without p19 (73% vs 86%, 42% vs 47%) (Fig. 5d). Nevertheless, given that p19-expressing plants exhibited marked differences in the severity of the leaf phenotype (Fig. 5b), we re-analyzed the results separating the three phenotypic groups (Fig. 5e). This new analysis showed that type I plants had the lowest mutation frequency (41% for *AP1* and 0% for *TT4*) while the mutation frequency dramatically increased for type III plants (100% for *AP1* and 86% for *TT4*), showing that the gene editing efficiency of CRISPR/Cas9 was strongly linked with the severity of p19-induced leaf phenotypes. To investigate this correlation, we quantified the levels of sgRNA and Cas9 transcripts in transgenic plants and found that type II and type III plants accumulated higher levels of sgRNA and Cas9 transcripts than control plants (transformed with CRISPR constructs lacking p19), while type I plants showed slightly lower levels than controls (Fig. 5f, g). The expression levels of miR168 was also quantified as an endogenous small RNA control and shown to be down-regulated in type I, but not types II and III plants (Fig. 5g).

### The viral p19-silencing suppressor improves the gene editing efficiency of CRISPR/Cas9 by interfering with the plant RNA-silencing pathway

**Fig. 6:**
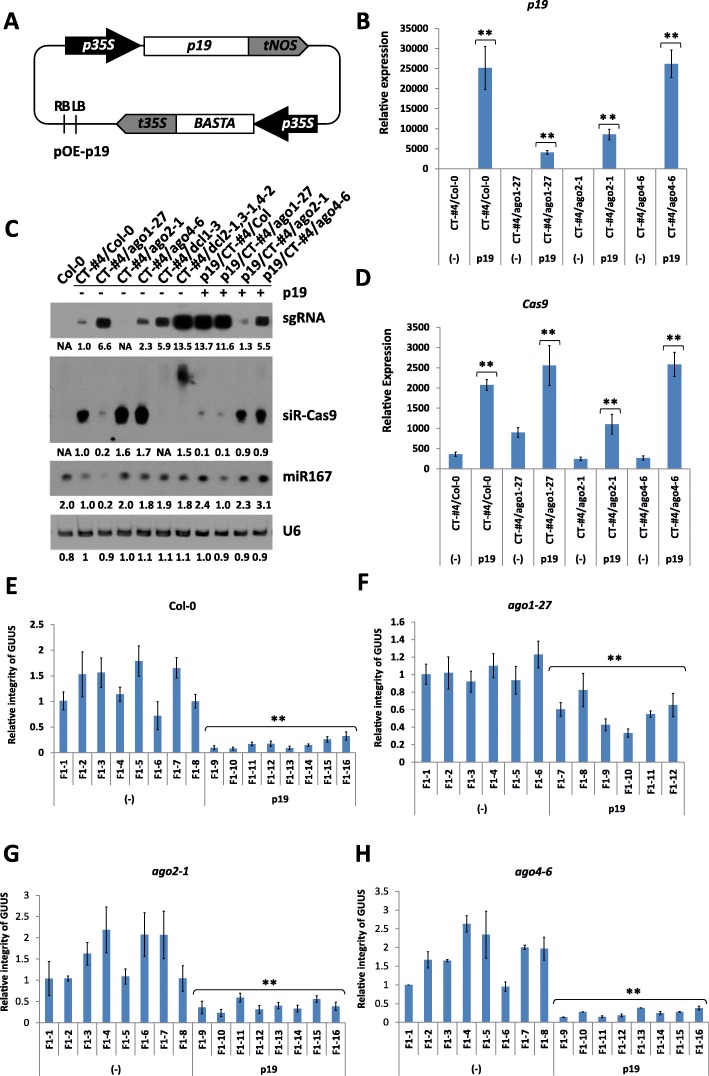
Establishing the role of p19 in CRISPR/Cas9 efficiency in Arabidopsis. **a** Schematic diagram of the p19 over-expression vector pOE-p19 used to transform CT-#4 lines of different mutant backgrounds. *BASTA*: bar resistance gene. **b** Relative p19 expression in pOE-p19 T2 transgenic plants of different CT-#4 backgrounds detected by quantitative RT-PCR. The columns indicate mean ± SD of three biological replicates. **c** sgRNA, siR-Cas9, and miR167 transcript levels in pOE-p19 T2 transgenic plants of different CT-#4 backgrounds detected by small RNA Northern blot. The U6 expression levels were used as internal reference. **d** Relative Cas9 expression levels in pOE-p19 T2 transgenic plants of different CT-#4 backgrounds detected by quantitative RT-PCR. The columns indicate mean ± SD of three biological replicates. **e**–**h** Relative integrity of the GUUS reporter gene in F1 hybrids of p19 overexpressing CT-#4 and GR-#16 lines in Col-0 (**e**), *ago1-27* (**f**), *ago2-1* (**g**) and *ago4-6* (**h**) backgrounds detect by real-time PCR. The columns indicate mean ± SD of three technical replicates. **Significant difference at *p* < 0.01 under two-tailed Student’s *t* test

To validate the positive effect of p19 in CRISPR/Cas9-mediated gene editing efficiency, we used the GUUS system described earlier. We first transformed CT-#4 lines containing a CRISPR/Cas9 cassette in the *ago1-27*, *ago2-1*, and *ago4-6* mutant backgrounds with a p19 ectopic expression vector (Fig. 6a). Mendelian segregation ratios were determined in T2 lines, and those showing single transgene integration were used for phenotypic observations. The expression levels of p19 were determined in the transgenic lines (Fig. 6b), and we noticed that homozygous p19 transgenic plants were usually sterile and exhibited stronger leaf developmental defects than heterozygotes (Additional file 1: Figure S2, S3). Therefore, only heterozygous T2 lines were subsequently used for hybridization with GR-#16 lines of the corresponding mutant backgrounds.

Analysis of the p19/CT-#4 lines showed that ectopic expression of p19 can further increase accumulation of the sgRNA and Cas9 transcripts in all of the tested mutant backgrounds (Fig. 6c, d). It has been proposed that p19 can protect viral genes from degradation by competing with the RISC complex for binding to siRNA duplexes [36]. To test this hypothesis, we determined the abundance of Cas9-derived siRNAs (siR-Cas9) in CT-#4 and p19/CT-#4 lines in different mutant backgrounds (Fig. 6c). The effect of p19 on siR-Cas9 was the opposite of that observed for Cas9 transcripts, with all p19/CT-#4 lines showing reduced siR-Cas9 levels. Interestingly, the size of siR-Cas9 transcripts was increased in *dcl2-1/dcl3-1/dcl4-2* mutants, suggesting that siR-Cas9 are processed to a final 20-22-nt size by one or more of the three DICER proteins. As mentioned above, expression of p19 resulted in a decrease in siR-Cas9 abundance in all tested samples, especially for Col-0; however, the small effect observed in the *ago1-27* background suggests that p19 acts by hampering the AGO1-directed degradation of Cas9 mRNA. Unlike siRNAs, endogenous miR167 levels were increased in the presence of p19 as previously reported (Fig. 6c) [30].

We subsequently tested the CRISPR/Cas9-mediated gene editing efficiency in p19/CE-#4 and GR-#16 F1 hybrids in different mutant backgrounds using the GUUS system (Fig. 6e–h). As expected, each F1 population showed an equal segregation ratio for plants containing or lacking the p19 expression cassette. The RI values for the GUUS gene were determined in both types of hybrids, and plants expressing p19 consistently showed lower RI values than their “non-p19” counterparts, consistent with our above results that p19 expression could improve the gene editing efficiency of the CRISPR/Cas9 system.

## Discussion

### The engineered CRISPR/Cas9 system is under negative regulation of the plant PTGS pathway

We hypothesized that ectopic expression of transcripts from the bacterial CRISPR/Cas9 system, especially the secondary structure-rich sgRNAs, may activate the plant RNA-silencing pathway. To test our hypothesis, we determined the mutagenesis efficiency of a previously optimized CRISPR/Cas9 system in Arabidopsis mutants for several genes involved in the two known plant RNA-silencing pathways: the post-transcriptional gene-silencing pathway (PTGS) and the transcriptional gene-silencing (TGS) pathway, also known as the RNA-directed DNA methylation (RdDM) pathway. Many important elements of the PTGS pathway are shared with the plant endogenous miRNA and siRNA pathways due to the requirement of 21–22-nt small RNA molecules to direct RNA cleavage. The TGS pathway is relatively distinct from the PTGS pathway, producing 24-nt small RNAs to direct transcriptional silencing through DNA methylation. Our results showed an increase in CRISPR/Cas9-mediated gene editing efficiency in PTGS pathway mutants, such as *ago1-27*, *dcl1-3*, and *dcl2-1/dcl3-1/dcl4-2*, but only a slight change in TGS pathway mutants, such as *dcl3-1* and *ago4-6ago6-4*, suggesting that the plant PTGS pathway is the major determinant limiting CRISPR/Cas9 efficiency (Fig. 1f). Consistent with this notion, we observed a moderate expression change of sgRNA and Cas9 transcripts in *CT-#4*/*ago4-6* compared to *CT-#4*/*ago1-27* and *CT-#4*/*dcl1-3* (Fig. 3b).

As a practical application of the observations from the PTGS mutants, we devised a practical approach including RNAi-based silencing modules for *AGO1* and *DCL1* in the CRISPR/Cas9 cassette. Although we obtained positive results with both genes using loss-of-function mutants, RNA interfering of *AGO1* proved to be more effective than *DCL1* mostly due to the unexpected observation that expression of double-stranded *DCL1* RNA molecules resulted in a strong increase in *DCL1* transcript levels in many transgenic lines.

### The viral silencing suppressor p19 can enhance the gene editing activity of CRISPR/Cas9 in stable transgenic lines in a dosage-dependent manner

The TBSV p19 forms a tail-to-tail homo-dimer preferentially binding 20-22-nt siRNA duplexes in a sequence-independent manner and prevents the assembly of the RISC complex [37]. The p19-mediated sequestration of siRNAs in virus-infected cells also blocks the spread of the mobile, systemic silencing signals by binding 21–25-nt siRNAs [25]. Although p19 has been widely adopted in transient expression systems to promote high levels of foreign gene expression, the developmental alterations caused by the ectopic expression of the protein have limited its application in stable transgenic plants. The p19-induced leaf alterations are probably caused by its interference with the plant endogenous siRNA and miRNA pathways [30, 38]. While p19 can bind many miRNAs, it does not bind miR168 that targets *AGO1* mRNA, thus reducing the accumulation of AGO1 [30, 39]. In this study, we observed reduced miR168 levels in type I transgenic plants expressing p19 together with the CRISPR/Cas9 cassette, but the expression levels recovered in type II and type III plants (Fig. 5g), suggesting a dosage-dependent regulation of miR168 by p19 during the interaction between virus and host. The two CRISPR elements, sgRNA and Cas9, were also regulated by p19 in a dosage-dependent manner. In type I transgenic plants with low p19 expression, showing a WT leaf phenotype, sgRNA and Cas9 transcript levels were lower than in plants transformed with the same CRISPR/Cas9 constructs without p19 (Fig. 5f, g). As the expression of p19 increased, the leaf phenotype became more accentuated (types II & III) and the transcript levels of sgRNA and Cas9 became higher (Fig. 3c). Considering that plants are continuously evolving counter measures to fight against viral suppressors by producing endogenous VSR recognition receptors [23], we think that the downregulation of miR168 and CRISPR/Cas9 transcripts observed in p19-low-expressing plants may be due to the triggering of the plant defense machinery while high p19 expression eventually overwhelms the defense response resulting in reduced transgene silencing.

### Practical applications of the p19-CRISPR/Cas9 and RNAi-*AGO1*-CRISPR/Cas9 systems for the identification of gene-edited plants

**Fig. 7:**
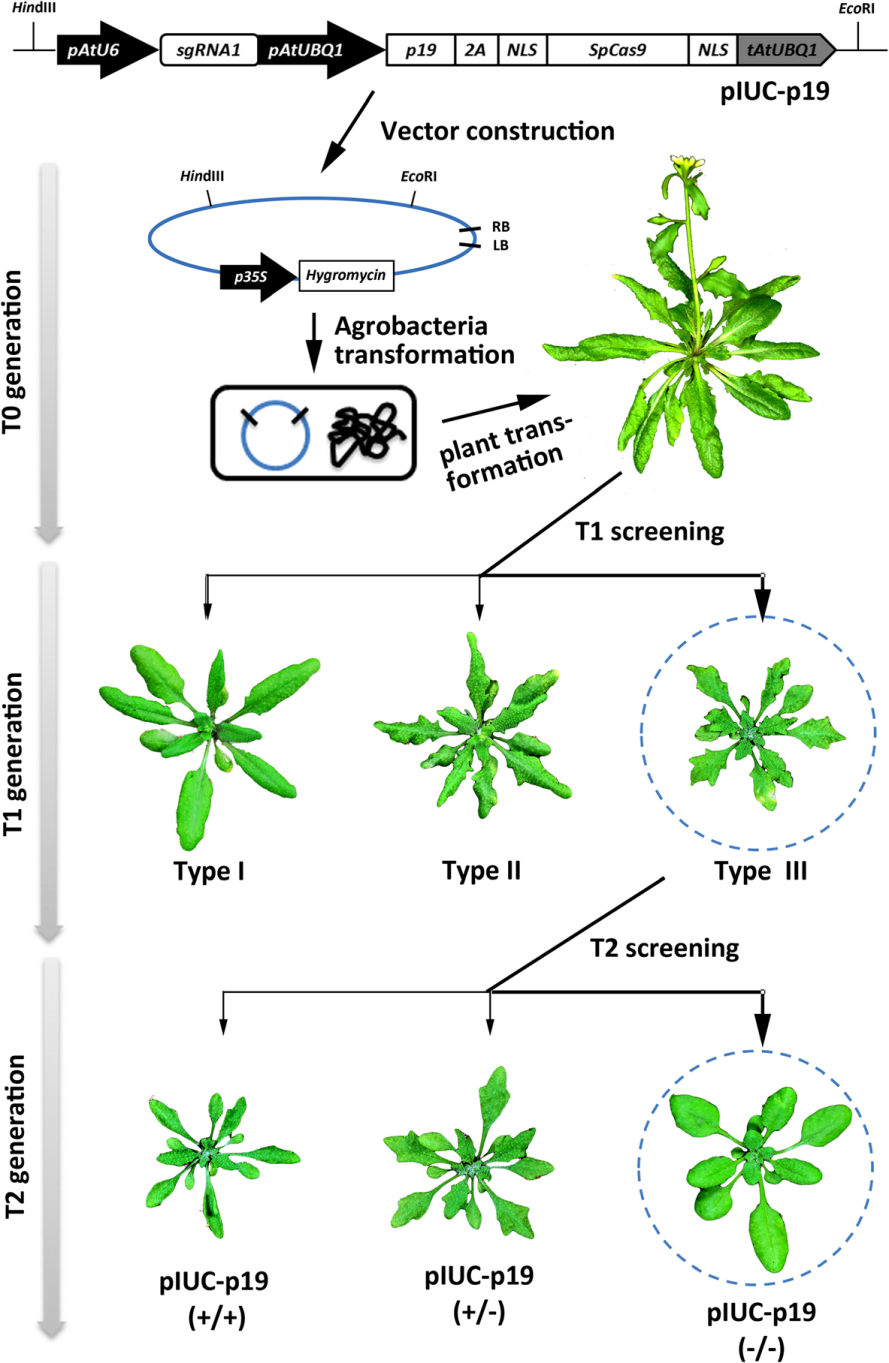
Workflow for the visual screening of stably inherited gene-edited mutants in Arabidopsis with the assistance of p19. The pIUC-p19 expression vector is used for Arabidopsis transformation via the Agrobacterium-mediated flower-dipping method. T1 plants are grouped into three types according to the severity of p19-induced leaf phenotypes. Type III plants will have a high probability of containing CRISPR/Cas9-induced mutations therefore analysis should be focused on these plants. T2 plants derived from gene-edited T1 seedlings are grouped again based on the p19-induced leaf phenotypes. Segregation of the inserted T-DNAs is easily recognized in this generation looking for wild-type looking plants. Screening for homozygous mutants without T-DNA inserts should be performed focusing on wild-type plants

The observations linking phenotype severity in the p19/CRISPR/Cas9 transgenic lines can have a very practical application to simplify the process of identifying CRISPR/Cas9-induced mutations in Arabidopsis, and perhaps other plant species (Fig. 7). Screening of T1 transgenic lines for mutations is arguably the most technically demanding and labor-intensive part in the production of CRISPR/Cas9-induced mutants and although a number of methods have been devised to screen T1 transgenic lines [40], they are technically demanding and costly. We have shown that T1 lines produced using our p19/CRISPR/Cas9 system have similar overall mutation frequencies to the non-p19 lines with no apparent benefits associated with the use of p19. Nevertheless, analysis of the data in conjunction with the observed developmental defects, showed extraordinarily high mutation rates on those lines showing strong phenotypes, increasing from 73% overall to 100% in type III plants for the *AP1* gene and from 42% overall to 86% in type III plants for *TT4*. Therefore, simple visual observation of T1 lines containing p19/CRISPR/Cas9 cassettes will provide two important benefits, (a) assurance that the lines are transgenic avoiding analysis of transformation escapes and (b) a vastly increased probability of finding CRISPR/Cas9 mutations on those lines. Although strong expression of p19 has important phenotypic effects on the T1 lines, this is not necessarily a disadvantage as phenotypic observations are usually performed on T1 plants. In fact, the strong p19-induced phenotype adds a third benefit to our approach since individuals with “non-serrated” leaves in the T2 generation will indicate that the p19 and CRISPR/Cas9-containing T-DNA has been segregated away. A similar strategy is possible incorporating the RNAi-*AGO1*-silencing module in the CRISPR/Cas9 cassette with AGO1-silencing phenotype severity being associated with CRISPR/Cas9 improved efficiency.

## Conclusion

In conclusion, we have shown for the first time that manipulation of the plant RNA-silencing pathway can lead to significant improvements in the gene editing efficiency of the bacterial-derived CRISRP/Cas system. In addition, we have developed two novel constructs to optimize the expression of CRISPR/Cas system in stable transgenic plants. Significant improvements in gene editing efficiency can be made by co-expressing either the viral-suppressor p19 or an *AGO1* RNA hairpin with CRISPR/Cas9. Our systems also provide a very easy method to identify plants with high frequency of gene mutations, thus simplifying the screening process. Considering that the RNA-silencing pathways are conserved in eukaryotes, we suggest that a similar strategy could be adopted in other species to simplify the identification of plants with CRISPR/Cas9-induced mutations.

## Methods

### Plant materials

*Arabidopsis thaliana* mutant lines in Columbia-0 (Col-0) ecotype for *ago1-27*, *ago2-1* (SALK_003380), *ago4-6/ago6-2* (SALK_071772/SALK_031553), *dcl1-3* (CS3176), *dcl2-1/dcl3-1/dcl4-2* (CS16391), and *dcl3-1* (SALK_005512) mutants were described previously [10, 12, 35, 41–43].

### Vector construction

The pTOE, pIUC-TT4, pIUC-AP1, CRISPR trigger, and GUUS reporter vector was constructed as described before [33, 44]. DNA fragments of TBSV p19 fused with 2A peptide were synthesized by GENEWIZ, China (Additional file 1: Note S). To produce the pIUC-p19 vectors, the synthesized p19-2A fragment was cloned into the *Nco*I site of pIUC-TT4 and pIUC-AP1 vectors to generate the pIUC-p19-TT4 and pIUC-p19-AP1 vector respectively. To allow the co-expression of an RNA interfering construct with the CRISPR/Cas9 systems, a gene fragment containing a *CaMV 35S* promoter, a multiple cloning site, and a *NOS* terminator in sequential was first amplified from the pTOE vector and subsequently inserted into the *Hin*dIII site of pIUC-TT4 to give an intermediate vector. Sense and antisense gene fragments of *AGO1*, *DCL1*, and the *PDK* intron were amplified separately using primer pairs listed in Additional file 1: Table S1. To generate the final pRNAi-Cas9-TT4 vectors, the paired sense and antisense gene fragments were then cloned into the *Sal*I and *Kpn*I site of the intermediate vector with a PDK intron in between using the Seamless Cloning and Assembly Kit (TransGen Biotech, China). To overexpress the p19 gene in CT-#4 lines, the coding sequences of p19 were first cloned into the *Sal*I and *Xba*I site of pTOE and subsequently introduced into the pCAMBIA3300 vector using *Hind*III and *Eco*RI (NEB, USA).

### Plant transformation and growth conditions

Agrobacterium-mediated transformation of *Arabidopsis thaliana* Columbia-0 (Col-0) with the binary vectors was performed using the floral dipping method as previously described [45]. Seed collected from the Agrobacterium-infected plants was sterilized with 2% sodium hypochlorite for 15 min and plated on Murashige and Skoog (MS0) medium containing 30 mg/L hygromycin or 25 mg/L phosphinothricin plus 50 mg/L carbenicillin to inhibit Agrobacterium growth. The resulting T1 plants were transplanted to soil after growing under long-day conditions (16-h light/8-h dark) at 22 °C for 2 weeks.

### Phenotype analysis for transgenic plants of the p19-coepressed CRISPR/Cas9 systems

For phenotype analysis of the *tt4* mutants, mature seeds of T1 plants were collected and registered according to the coloration of seed coats. Plant developed uniformly brown-colored seeds were designated as wild type, those grew uniformly yellow-colored seeds were designated as mutant, and those produced seed of both colors are designated as chimera. For phenotype analysis of the *ap1* mutants, phenotype analysis was performed during the flowering times. Compared to wild-type plants, *ap1* mutants developed uniformly petal-less flowers, while the chimera produce partially petal-less flowers and partially wild-type flowers. For phenotype analysis of p19 transgenic lines, the p19-induced leaf phenotypes were also classified into three types according to the severity. Type I plants have normal wild-type leaves, type II plants develop curly leaves, and type III plants grow curly and serrated leaves.

### Northern blot for sgRNA, miRNAs, and Cas9 gene in transgenic plants

RNA samples were extracted from the transgenic plants using the Trizol® reagents (Invitrogen, USA). For small RNA detection, 20 μg of total RNAs or 10 μg small RNAs was resolved by 15% PAGE in 1×Tris-borate-EDTA. The 3′biotin-labeled DNA oligos, anti-sgRNA, anti-miR167, and anti-miR168, were synthesized by Invitrogen, USA. Hybridization and membrane wash were performed under 42 °C. Blot signals were detected using the Chemiluminescent Nucleic Acid Detection Module Kit (Thermo Fisher, USA). For Cas9 detection, 30 μg total RNA was resolved by 1% Formaldehyde-agarose gel. Biotin-labeled probes used for Cas9 detection were synthesized by in vitro transcription of a 286 bp Cas9 fragment using the North2South™ Complete Biotin Random Prime Labeling and Detection Kit (Thermo Fisher, USA). Gel transfer, hybridization, and membrane wash were performed as described before. The blot signals were detected using the Chemiluminescent Nucleic Acid Detection Module Kit (Thermo Fisher, USA).

### Real-time PCR

For quantitative real-time PCR, total RNA was treated with DNase I (TaKaRa, Japan) followed by phenol/chloroform extraction to remove DNA contamination. Approximately 1 μg of purified RNAs was used for first-strand complementary DNA synthesis using PrimeScript Reverse Transcriptase (TaKaRa, Japan) with oligo(dT) primers. Real-time PCR was performed using iQSYBR Green Supermix (Bio-Rad) with primer pairs listed in Additional file 1: Table S1. Relative transcript levels were determined for each sample by normalizing them to *ACTIN* according to the ∆∆Ct method [46].

For calculating the relative integrity (RI) value of GUUS and TT4 gene, DNA samples were prepared using CTAB methods. Approximately 50 ng DNA was used for PCR amplification. Real-time PCR was performed using iQSYBR Green Supermix (Bio-Rad, USA) with primer pairs listed in Additional file 1: Table S1. The RI value of GUUS gene were determined by normalizing the Ct Value of U fragment to S fragment according to the ∆∆Ct method and the RI value of TT4 gene were calculated similarly by normalizing the Ct Value of sgRNA-targeted regions to non-targeted regions using primer pairs listed in Additional file 1: Table S1.

### Western blot

Total protein were extracted from 150-mg floral tissue of the transgenic plants and resolved on 12% SDS-PAGE. Immunoblot was performed using anti-2A antibody at a dilution of 1/1000 (Merck, Germany). The blot signals were detected using Pierce™ ECL Western Blotting Substrate (Thermo Fisher, USA).

## Additional file

Additional file 1: **Figure S1.** A schematics showing the sequences of the sgRNA targeting site and FP1 in GUUS reporter gene. **Figure S2.** Phenotypes of the pOE-p19 T2 lines in CT-#4 of mutant backgrounds. **Figure S3.** Segregation of pOE-p19 transgenic plants in T2 generation. Table S1. Primers used in this study. Note S. Sequences of the TBSV p19-2A, pRNAi-Cas9 cassettes, the pIUC-p19 cassettes, and the GUUS cassette. (PDF 570 kb)
