# Supplementary material for: Manipulating plant RNA-silencing pathways to improve the gene editing efficiency of CRISPR/Cas9 systems
Source: Genome Biol. 2018 Sep 28;19:149. doi: 10.1186/s13059-018-1529-7 (PMC6161460; doi:10.1186/s13059-018-1529-7)
Supplement: Supplementary file 1 — Figure S1. A schematics showing the sequences of the sgRNA targeting site and FP1 in GUUS reporter gene. Figure S2. Phenotypes of the pOE-p19 T2 lines in CT-#4 of mutant backgrounds. Figure S3. Segregation of pOE-p19 transgenic plants in T2 generation. Table S1. Primers used in this study. Note S. Sequences of the TBSV p19-2A, pRNAi-Cas9 cassettes, the pIUC-p19 cassettes, and the GUUS cassette. (PDF 570 kb) [file 13059_2018_1529_MOESM1_ESM.pdf]

Additional file 1: Figure S1. A schematic showing the sequences of the sgRNA targeting site and FP1 in GUUS reporter gene.

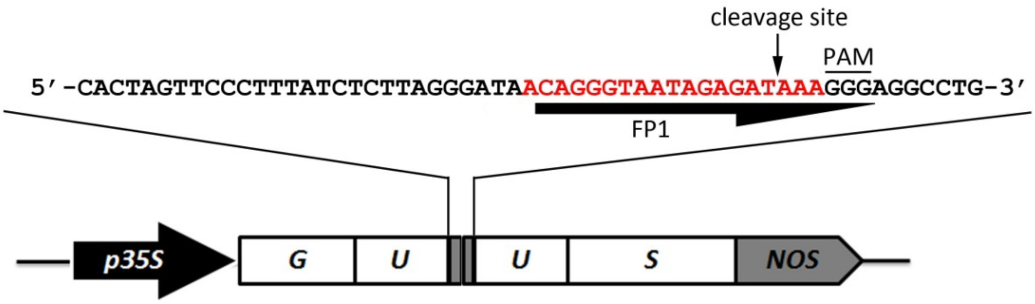

Additional file 1: Figure S2. Phenotypes of the pOE-p19 T2 lines in CT-#4 of mutant backgrounds. Bar=1 cm.

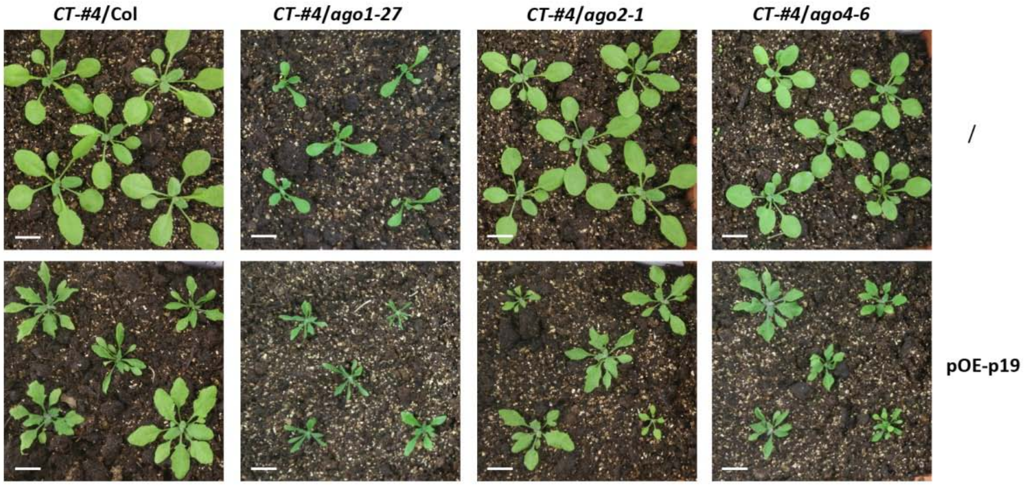

Additional file 1: Figure S3. Segregation of pOE-p19 transgenic plants in T2 generation. Bar=1cm

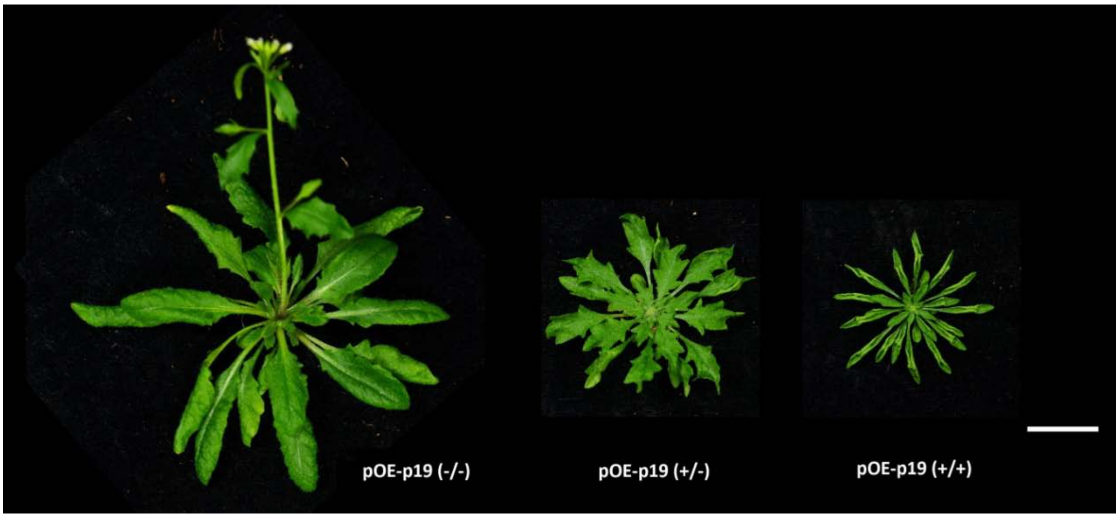

Additional file 1: Table S1. Primers used in this study

	Primer	Sequences 5'-3'
pIUC-p19	p19-F-SalI	AGGTCGACCATGGAACGAGCTATACAAGGAAACG
	2A-R-BsaI	TTTGGTCTCCCATGCCAGGTCCAGGGTTCTCCT
pRNAi-Cas9	PDK intron-F	CCAATTGGTAAGGAAATAATTATTTTCTTTTTTCCTTTTA
	PDK intron-R	CAACTGTAATCAATCCAAATGTAAGATCAATGATAACACA
	AGO1 5'UTR-36F	GAGAGGACACTGCAGGTCGACTCGTTTGTTCGGAGTTAGAG
	AGO1 5'UTR-166R	ATTATTTCCTTACCAATTGGAAAAGCAATCTCTCGCCGGA
	AGO1 5'UTR reverse-166F	ATTTGGATTGATTACAGTTGAAAAGCAATCTCTCGCCGGA
	AGO1 5'UTR reverse-36R	TGAACGCCTGAGCTCGGTACCTCGTTTGTTCGGAGTTAGAG
	AGO1-926F	TTTGGAGAGGACACGCTCGAGACAATCAGATGCCCCACAGG
	AGO1-1520R	ATTATTTCCTTACCAATTGGTACCGCTGGCCTTCAACAAT
	AGO1 reverse-1520F	ATTTGGATTGATTACAGTTGGTACCGCTGGCCTTCAACAA
	AGO1 reverse-926R	CATTAAAGCAGGACTCTAGAACAATCAGATGCCCCACAGG
	DCL1 3'UTR-F	GAGAGGACACTGCAGGTCGACTCTTTTACTCTCTTCAACGA
	DCL1 3'UTR-R	ATTATTTCCTTACCAATTGGACCAATTAAACTCGAATAAC
	DCL1 3'UTR reverse-F	ATTTGGATTGATTACAGTTGACCAATTAAACTCGAATAAC
	DCL1 3'UTR reverse-R	TGAACGCCTGAGCTCGGTACCTCTTTTACTCTCTTCAACGA
	DCL1 CDS-309F	GAGAGGACACTGCAGGTCGACTGGGTTGCAAAAGAATGGCG
	DCL1 CDS-691R	ATTATTTCCTTACCAATTGGCTGCCTTGACATCGCATTCG
	DCL1 CDS reverse-691F	ATTTGGATTGATTACAGTTGCTGCCTTGACATCGCATTCG
	DCL1 CDS reverse-309R	TGAACGCCTGAGCTCGGTACCTGGGTTGCAAAAGAATGGCG
Probes	anti-sgR	AGCACCGACTCGGTGCCACT-3'biotin
	anti-U6	TCATCCTTGCGCAGGGGCCA-3'biotin
	anti-167	TAGATCATGCTGGCAGCTTCA-3'biotin
	anti-168	TTCCCGACCTGCACCAAGCGA-3'biotin
Realtime PCR	Cas9-92F	GGTCGGTATCCACGGAGTCCCAGC
	Cas9-471R	GATGGGGTGCCGCTCGTGCTTC
	p19-380F	GCGAACTCTTCAGCATCTCT
	p19-483R	AGGGCATCCTCTTGATACATTAC
	DCL1-2022F	GGTTGCTGCTTTGGTTCTTC
	DCL1-2123R	TTCATCTCCTGGCTGTTATTGT
	AGO1-1548F	CACTGCTTTGCTGAAGGTTAC

	AGO1-1693R	CCTCAACAGAAGCCAGAGAA
	Actin-F	TGGCATCAYACTTTCTACAA
	Actin-R	CCACCACTDAGCACAATGTT
RI Value	TT4-65F	AGAGAGCTGATGGACCTGC
	TT4-296R	AATTGTCGACTTGTCGCCT
	Actin-GF	GACTCTGGAGATGGTGTGAG
	Actin-R	CCACCACTDAGCACAATGTT
	GUUS-FP1	AACAGGGTAATAGAGATAAAG
	GUUS-RP1	TTTGCCGTAATGAGTGACC
	GUUS-FP0	CTGAAGAGATGCTCGACTG
	GUUS-RP0	CAGCTCTTTAATCGCCTGTA

Note S. Sequences of the TBSV p19-2A, pRNAi-Cas9 cassettes, the pIUC-p19 cassettes and the GUUS cassette.

> TBSV p19-2A
ATGGAACGAGCTATACAAGGAAACGACGCTAGGGAACAAGCTAACAGTGAACGTTGGGATGGAGGATCAG
GAGGAACCACTTCTCCCTTCAAACTTCCTGACGAAAGTCCGAGTTGGACTGAGTGGCGGCTACATAACGAT
GAGACTAATTCGAATCAAGATAATCCCCTTGGTTTCAAGGAAAGCTGGGGTTTCGGGAAAGTTGTATTTAAG
AGATATCTCAGATACGACAGGACGGAGGCTTCACTGCACAGAGTCCTTGGATCTTGGACGGGAGATTCGGT
TAACTATGCAGCATCTCGATTTTTCGGTTTCGACCAGATCGGATGTACCTATAGTATTCGGTTTCGAGGAGTTA
GTATCACCGTTTCTGGAGGGTCGCGAACTCTTCAGCATCTCTGTGAGATGGCAATTCGGTCTAAGCAAGAAC
TGCTACAGCTTGCCCCAATCGAAGTGGAAAGTAATGTATCAAGAGGATGCCCTGAAGGTACTGAAACCTTCG
AAAAAGAAAGCGAGGGAAGCGGAGCTACTAACTTCAGCCTGCTGAAGCAGGCTGGTGACGTGGAGGAGA
ACCCTGGACCT
“_” : 2A-peptide; “ATG“: Start codon

>p35s-PDK-tNOS
AAGCTTAGTCAAAGATTCAAATAGAGGACCTAACAGAACTCGCCGTAAAGACTGGCGAACAGTTCATACAG
AGTCTCTTACGACTCAATGACAAGAAGAAAATCTTCGTCAACATGGTGGAGCACGACACGCTTGTCTACTCC
AAAAATATCAAAGATACAGTCTCAGAAGACCAAAGGGCAATTGAGACTTTTCAACAAAGGGTAATATCCGG
AAACCTCCTCGGATTCCATTGCCCAGCTATCTGTCACTTTATTGTGAAGATAGTGGAAAAGGAAGGTGGCTC
CTACAAATGCCATCATTGCGATAAAGGAAAGGCCATCGTTGAAGATGCCTCTGCCGACAGTGGTCCCAAAGA
TGGACCCCCACCCACGAGGAGCATCGTGGAAAAAGAAGACGTTCCAACCACGTCTTCAAAGCAAGTGGAT
TGATGTGATATCTCCACTGACGTAAGGGATGACGCACAATCCCACTATCCTTCGCAAGACCCTTCCTCTATATA
AGGAAGTTCATTTCATTTGGAGAGGACACTGCAGGTCGACCCAATTGGTAAGGAAATAATTATTTTCTTTTTT
CCTTTTAGTATAAAATAGTTAAGTGATGTTAATTAGTATGATTATAATAATATAGTTGTTATAATTGTGAAAAAAT
AATTTATAAATATATTGTTTACATAAACAACATAGTAATGTAAAAAAATATGACAAGTGATGTGTAAGACGAAG
AAGATAAAAGTTGAGAGTAAGTATATTATTTTTAATGAATTTGATCGAACATGTAAGATGATATACTAGCATTAA
TATTTGTTTTAATCATAATAGTAATTCTAGCTGGTTTGATGAATTAAATATCAATGATAAAATACTATAGTAAAAA
TAAGAATAAATAAATTAAAATAATATTTTTTTATGATTAATAGTTTATTATATAATTAAATATCTATACCATTACTAA
ATATTTTAGTTTAAAAGTTAATAAATATTTTGTTAGAAATTCCAATCTGCTTGTAATTTATCAATAAACAAAATAT
TAAATAACAAGCTAAAGTAACAAATAATATCAAACTAATAGAAACAGTAATCTAATGTAACAAAACATAATCTA
ATGCTAATATAACAAAGCGCAAGATCTATCATTTTATATAGTATTATTTTCAATCAACATTCTTATTAATTTCTAAA
TAATACTTGTAGTTTTATTAACTTCTAAATGGATTGACTATTAATTAAATGAATTAGTCGAACATGAATAAACAA
GGTAACATGATAGATCATGTCATTGTGTTATCATTGATCTTACATTTGGATTGATTACAGTTGCTGGTACCGAG
CTCAGGCGTTCAAACATTTGGCAATAAAGTTTCTTAAGATTGAATCCTGTTGCCGGTCTTGCGATGATTATCAT
ATAATTTCTGTTGAATTACGTTAAGCATGTAATAATTAACATGTAATGCATGACGTTATTTATGAGATGGGTTTT
TATGATTAGAGTCCCGCAATTATACATTTAATACGCGATAGAAAACAAAATATAGCGCGCAAACTAGGATAAAT
TATCGCGCGCGGTGTCATCTATGTTACTAGATCGGGAATTGCCAATAGCTT
“_” : PDK intron

>pAtU6-sgR-IR
TAGCTTCATTCGGAGTTTTTGTATCTTGTTTCATAGTTTGTCCCAGGATTAGAATGATTAGGCATCGAACCTTC
AAGAATTTGATTGAATAAAACATCTTCATTCTTAAGATATGAAGATAATCTTCAAAAGGCCCCTGGGAATCTG
AAAGAAGAGAAGCAGGCCCATTTATATGGGAAAGAACAATAGTATTTCTTATATAGGCCCATTTAAGTTGAA

AACAATCTTCAAAAGTCCCACATCGCTTAGATAAGAAAACGAAGCTGAGTTTATATACAGCTAGAGTCGAAG
TAGTGATT**GAACAGGGTAATAGAGATAAA**GTTTTAGAGCTAGAAATAGCAAGTTAAAATAAGGCTAGTCCG
TTATCAACTTGAAAAAGTGGCACCGAGTCGGTGCTTTTTTGTTTTAGAGCTAGAAATAGCAAGTTAAAATAA
GGCTAGTCCGTAGCGCGTGCGCCAATTCTGCAGACAAATGGCCCCGGG
"_" : sgR-IR; "**Bold**": IR guide sequences

> pAtUBQ-NLS-SpCas9-BPNLS-tUBQ
CCCGGGATATTTCACAAATTGAACATAGACTACAGAATTTTAGAAAACAAACTTTCTCTCTCTTATCTCACCTT
TATCTTTTAGAGAGAAAAAGTTCGATTTCCGGTTGACCGGAATGTATCTTTGTTTTTTTTGTTTTGTAACATAT
TTCGTTTTCCGATTTAGATCGGATCTCCTTTTCCGTTTTGTCGGACCTTCTTCCGGTTTATCCGGATCTAATAAT
ATCCATCTTAGACTTAGCTAAGTTTGGATCTGTTTTTTGGTTAGCTCTTGTCAATCGCCTCATCATCAGCAAGA
AGGTGAAATTTTTGACAAATAAATCTTAGAATCATGTAGTGTCTTTGGACCTTGGGAATGATAGAAACGATTT
GTTATAGCTACTCTATGTATCAGACCCTGACCAAGATCCAACAATCTCATAGGTTTTGTGCATATGAAACCTTC
GACTAACGAGAAGTGGTCTTTTAATGAGAGAGATATCTAAAATGTTATCTTAAAAGCCCACTCAAATCTCAAG
GCATAAGGTAGAAATGCAAATTTGGAAAGTGGGCTGGGCCTTTTGTGGTAAAGGCCTGTAACCTAGCCCAA
TATTAGCAAAACCCTAGACGCGTACATTGACATATATAAACCCGCCTCCTCCTTGTTTAGGGTTTCTACGTGAG
AGAGACGAAACACAAACCATGGACTATAAGGACCACGACGGAGACTACAAGGATCATGATATTGATTACAA
AGACGATGACGATAAGATGGCCCCAAAGAAGAAGCGGAAGGTCGGTATCCACGGAGTCCCAGCAGCCGAC
AAGAAGTACAGCATCGGCCTGGACATCGGCACCAACTCTGTGGGCTGGGCCGTGATCACCGACGAGTACAA
GGTGCCCAGCAAGAAATTCAAGGTGCTGGGCAACACCGACCGGCACAGCATCAAGAAGAACCTGATCGGA
GCCCTGCTGTTCGACAGCGGCGAAACAGCCGAGGCCACCCGGCTGAAGAGAACCGCCAGAAGAAGATAC
ACCAGACGGAAGAACCGGATCTGCTATCTGCAAGAGATCTTCAGCAACGAGATGGCCAAGGTGGACGACA
GCTTCTTCCACAGACTGGAAGAGTCCTTCCTGGTGGAAGAGGATAAGAAGCACGAGCGGCACCCCATCTTC
GGCAACATCGTGGACGAGGTGGCCTACCACGAGAAGTACCCCACCATCTACCACCTGAGAAAGAAACTGGT
GGACAGCACCGACAAGGCCGACCTGCGGCTGATCTATCTGGCCCTGGCCCACATGATCAAGTTCCGGGGCC
ACTTCCTGATCGAGGGCGACCTGAACCCCGACAACAGCGACGTGGACAAGCTGTTCATCCAGCTGGTGCAG
ACCTACAACCAGCTGTTCGAGGAAAACCCCATCAACGCCAGCGGCGTGGACGCCAAGGCCATCCTGTCTGC
CAGACTGAGCAAGAGCAGACGGCTGGAAAATCTGATCGCCCAGCTGCCCGGCGAGAAGAAGAATGGCCT
GTTCGGCAACCTGATTGCCCTGAGCCTGGGCCTGACCCCCAACTTCAAGAGCAACTTCGACCTGGCCGAGG
ATGCCAAACTGCAGCTGAGCAAGGACACCTACGACGACGACCTGGACAACCTGCTGGCCCAGATCGGCGA
CCAGTACGCCGACCTGTTTCTGGCCGCCAAGAACCTGTCCGACGCCATCCTGCTGAGCGACATCCTGAGAGT
GAACACCGAGATCACCAAGGCCCCCCTGAGCGCCTCTATGATCAAGAGATACGACGAGCACCACCAGGACC
TGACCCTGCTGAAAGCTCTCGTGCGGCAGCAGCTGCCTGAGAAGTACAAAGAGATTTTCTTCGACCAGAGC
AAGAACGGCTACGCCGGCTACATTGACGGCGGAGCCAGCCAGGAAGAGTTCTACAAGTTCATCAAGCCCAT
CCTGGAAAAGATGGACGGCACCGAGGAACTGCTCGTGAAGCTGAACAGAGAGGACCTGCTGCGGAAGCA
GCGGACCTTCGACAACGGCAGCATCCCCCACCAGATCCACCTGGGAGAGCTGCACGCCATTCTGCGGCGGC
AGGAAGATTTTTACCCATTCCTGAAGGACAACCGGGAAAAGATCGAGAAGATCCTGACCTTCCGCATCCCCT
ACTACGTGGGCCCTCTGGCCAGGGGAAACAGCAGATTCGCCTGGATGACCAGAAAGAGCGAGGAAACCAT
CACCCCCTGGAACTTCGAGGAAGTGGTGGACAAGGGCGCTTCCGCCCAGAGCTTCATCGAGCGGATGACC
AACTTCGATAAGAACCTGCCCAACGAGAAGGTGCTGCCCAAGCACAGCCTGCTGTACGAGTACTTCACCGT
GTATAACGAGCTGACCAAAGTGAAATACGTGACCGAGGGAATGAGAAAGCCCGCCTTCCTGAGCGGCGAG
CAGAAAAAGGCCATCGTGGACCTGCTGTTCAAGACCAACCGGAAAGTGACCGTGAAGCAGCTGAAAGAG
GACTACTTCAAGAAAATCGAGTGCTTCGACTCCGTGGAAATCTCCGGCGTGGAAGATCGGTTCAACGCCTC
CCTGGGCACATACCACGATCTGCTGAAAATTATCAAGGACAAGGACTTCCTGGACAATGAGGAAAACGAGG

ACATTCTGGAAGATATCGTGCTGACCCTGACACTGTTTGAGGACAGAGAGATGATCGAGGAACGGCTGAAA
ACCTATGCCCACCTGTTCGACGACAAAGTGATGAAGCAGCTGAAGCGGCGGAGATACACCGGCTGGGGCA
GGCTGAGCCGGAAGCTGATCAACGGCATCCGGGACAAGCAGTCCGGCAAGACAATCCTGGATTTCCTGAA
GTCCGACGGCTTCGCCAACAGAAACTTCATGCAGCTGATCCACGACGACAGCCTGACCTTTAAAGAGGACA
TCCAGAAAGCCCAGGTGTCCGGCCAGGGCGATAGCCTGCACGAGCACATTGCCAATCTGGCCGGCAGCCC
CGCCATTAAGAAGGGCATCCTGCAGACAGTGAAGGTGGTGGACGAGCTCGTGAAAGTGATGGGCCGGCAC
AAGCCCGAGAACATCGTGATCGAAATGGCCAGAGAGAACCAGACCACCCAGAAGGGACAGAAGAACAGC
CGCGAGAGAATGAAGCGGATCGAAGAGGGCATCAAAGAGCTGGGCAGCCAGATCCTGAAAGAACACCCC
GTGGAAAACACCCAGCTGCAGAACGAGAAGCTGTACCTGTACTACCTGCAGAATGGGCGGGATATGTACGT
GGACCAGGAACTGGACATCAACCGGCTGTCCGACTACGATGTGGACCATATCGTGCCTCAGAGCTTTCTGA
AGGACGACTCCATCGACAACAAGGTGCTGACCAGAAGCGACAAGAACCGGGGCAAGAGCGACAACGTGC
CCTCCGAAGAGGTCGTGAAGAAGATGAAGAACTACTGGCGGCAGCTGCTGAACGCCAAGCTGATTACCCA
GAGAAAGTTCGACAATCTGACCAAGGCCGAGAGAGGCGGCCTGAGCGAACTGGATAAGGCCGGCTTCATC
AAGAGACAGCTGGTGGAAACCCGGCAGATCACAAAGCACGTGGCACAGATCCTGGACTCCCGGATGAACA
CTAAGTACGACGAGAATGACAAGCTGATCCGGGAAGTGAAAGTGATCACCCTGAAGTCCAAGCTGGTGTCC
GATTTCCGGAAGGATTTCCAGTTTTACAAAGTGCGCGAGATCAACAACTACCACCACGCCCACGACGCCTAC
CTGAACGCCGTCGTGGGAACCGCCCTGATCAAAAAGTACCCTAAGCTGGAAAGCGAGTTCGTGTACGGCG
ACTACAAGGTGTACGACGTGCGGAAGATGATCGCCAAGAGCGAGCAGGAAATCGGCAAGGCTACCGCCAA
GTACTTCTTCTACAGCAACATCATGAACTTTTTCAAGACCGAGATTACCCTGGCCAACGGCGAGATCCGGAA
GCGGCCTCTGATCGAGACAAACGGCGAAACCGGGGAGATCGTGTGGGATAAGGGCCGGGATTTTGCCACC
GTGCGGAAAGTGCTGAGCATGCCCCAAGTGAATATCGTGAAAAAGACCGAGGTGCAGACAGGCGGCTTCA
GCAAAGAGTCTATCCTGCCCAAGAGGAACAGCGATAAGCTGATCGCCAGAAAGAAGGACTGGGACCCTAA
GAAGTACGGCGGCTTCGACAGCCCCACCGTGGCCTATTCTGTGCTGGTGGTGGCCAAAGTGGAAAAGGGC
AAGTCCAAGAAACTGAAGAGTGTGAAAGAGCTGCTGGGGATCACCATCATGGAAAGAAGCAGCTTCGAGA
AGAATCCCATCGACTTTCTGGAAGCCAAGGGCTACAAAGAAGTGAAAAAGGACCTGATCATCAAGCTGCCT
AAGTACTCCCTGTTCGAGCTGGAAAACGGCCGGAAGAGAATGCTGGCCTCTGCCGGCGAACTGCAGAAGG
GAAACGAACTGGCCCTGCCCTCCAAATATGTGAACTTCCTGTACCTGGCCAGCCACTATGAGAAGCTGAAGG
GCTCCCCCGAGGATAATGAGCAGAAACAGCTGTTTGTGGAACAGCACAAGCACTACCTGGACGAGATCATC
GAGCAGATCAGCGAGTTCTCCAAGAGAGTGATCCTGGCCGACGCTAATCTGGACAAAGTGCTGTCCGCCTA
CAACAAGCACCGGGATAAGCCCATCAGAGAGCAGGCCGAGAATATCATCCACCTGTTTACCCTGACCAATCT
GGGAGCCCCTGCCGCCTTCAAGTACTTTGACACCACCATCGACCGGAAGAGGTACACCAGCACCAAAGAG
GTGCTGGACGCCACCCTGATCCACCAGAGCATCACCGGCCTGTACGAGACACGGATCGACCTGTCTCAGCT
GGGAGGCGACAAGAGAACCGCCGACGGAAGCGAGTTCGAGAGCCCTAAGAAAAAGCGGAAAGTGGAGT
AAGGATCCAGAGACTCTTATCAAGAATCCCATCTCTTGCTTGCTTTTTTTTGTTGCTTCCCTTTGATAGGGTTT
GTTTTTCTTGTTTCAGTGACTTTCTATGTTAAAAGATAATGTCAGTAAAAGGATTTGGTTTTCTATTATTCTGAA
TCGATTACGGAAGATTCTTGCTTAATTCCAATCTATACAAGTATCGTGAAATAATGACCGTTTATGTGGTACC
“_” : SpCas9; “_” : NLS-BPNLS; “ATG“: Start codon; “TAA“: Stop codon

>pAtUBQ-p19-2A-NLS-SpCas9-NLS-tUBQ
CCCGGGATATTTCACAAATTGAACATAGACTACAGAATTTTAGAAAACAAACTTTCTCTCTCTTATCTCACCTT
TATCTTTTAGAGAGAAAAAGTTCGATTTCCGGTTGACCGGAATGTATCTTTGTTTTTTTTGTTTTGTAACATAT
TTCGTTTTCCGATTTAGATCGGATCTCCTTTTCCGTTTTGTCGGACCTTCTTCCGGTTTATCCGGATCTAATAAT
ATCCATCTTAGACTTAGCTAAGTTTGGATCTGTTTTTTGGTTAGCTCTTGTCAATCGCCTCATCATCAGCAAGA
AGGTGAAATTTTTGACAAATAAATCTTAGAATCATGTAGTGTCTTTGGACCTTGGGAATGATAGAAACGATTT

GTTATAGCTACTCTATGTATCAGACCCTGACCAAGATCCAACAATCTCATAGGTTTTGTGCATATGAAACCTTC
GACTAACGAGAAGTGGTCTTTTAATGAGAGAGATATCTAAAATGTTATCTTAAAAGCCCACTCAAATCTCAAG
GCATAAGGTAGAAATGCAAATTTGGAAAGTGGGCTGGGCCTTTTGTGGTAAAGGCCTGTAACCTAGCCCAA
TATTAGCAAAACCCTAGACGCGTACATTGACATATATAAACCCGCCTCCTCCTTGTTTAGGGTTTCTACGTGAG
AGAGACGAAACACAAACCATGGAACGAGCTATACAAGGAAACGACGCTAGGGAACAAGCTAACAGTGAAC
GTTGGGATGGAGGATCAGGAGGAACCACTTCTCCCTTCAAACTTCCTGACGAAAGTCCGAGTTGGACTGAG
TGGCGGCTACATAACGATGAGACTAATTCGAATCAAGATAATCCCCTTGGTTTCAAGGAAAGCTGGGGTTTC
GGGAAAGTTGTATTTAAGAGATATCTCAGATACGACAGGACGGAGGCTTCACTGCACAGAGTCCTTGGATC
TTGGACGGGAGATTCGGTTAACTATGCAGCATCTCGATTTTTCGGTTTCGACCAGATCGGATGTACCTATAGT
ATTCGGTTTCGAGGAGTTAGTATCACCGTTTCTGGAGGGTCGCGAACTCTTCAGCATCTCTGTGAGATGGCA
ATTCGGTCTAAGCAAGAACTGCTACAGCTTGCCCCAATCGAAGTGGAAAGTAATGTATCAAGAGGATGCCCT
GAAGGTACTGAAACCTTCGAAAAAGAAAGCGAGGGAAGCGGAGCTACTAACTTCAGCCTGCTGAAGCAG
GCTGGTGACGTGGAGGAGAACCCTGGACCTGGCATGGACTATAAGGACCACGACGGAGACTACAAGGATC
ATGATATTGATTACAAAGACGATGACGATAAGATGGCCCCAAAGAAGAAGCGGAAGGTCGGTATCCACGGA
GTCCCAGCAGCCGACAAGAAGTACAGCATCGGCCTGGACATCGGCACCAACTCTGTGGGCTGGGCCGTGA
TCACCGACGAGTACAAGGTGCCCAGCAAGAAATTCAAGGTGCTGGGCAACACCGACCGGCACAGCATCAA
GAAGAACCTGATCGGAGCCCTGCTGTTCGACAGCGGCGAAACAGCCGAGGCCACCCGGCTGAAGAGAAC
CGCCAGAAGAAGATACACCAGACGGAAGAACCGGATCTGCTATCTGCAAGAGATCTTCAGCAACGAGATG
GCCAAGGTGGACGACAGCTTCTTCCACAGACTGGAAGAGTCCTTCCTGGTGGAAGAGGATAAGAAGCACG
AGCGGCACCCCATCTTCGGCAACATCGTGGACGAGGTGGCCTACCACGAGAAGTACCCCACCATCTACCAC
CTGAGAAAGAAACTGGTGGACAGCACCGACAAGGCCGACCTGCGGCTGATCTATCTGGCCCTGGCCCACAT
GATCAAGTTCCGGGGCCACTTCCTGATCGAGGGCGACCTGAACCCCGACAACAGCGACGTGGACAAGCTG
TTCATCCAGCTGGTGCAGACCTACAACCAGCTGTTCGAGGAAAACCCCATCAACGCCAGCGGCGTGGACGC
CAAGGCCATCCTGTCTGCCAGACTGAGCAAGAGCAGACGGCTGGAAAATCTGATCGCCCAGCTGCCCGGC
GAGAAGAAGAATGGCCTGTTCGGCAACCTGATTGCCCTGAGCCTGGGCCTGACCCCCAACTTCAAGAGCA
ACTTCGACCTGGCCGAGGATGCCAAACTGCAGCTGAGCAAGGACACCTACGACGACGACCTGGACAACCT
GCTGGCCCAGATCGGCGACCAGTACGCCGACCTGTTTCTGGCCGCCAAGAACCTGTCCGACGCCATCCTGC
TGAGCGACATCCTGAGAGTGAACACCGAGATCACCAAGGCCCCCCTGAGCGCCTCTATGATCAAGAGATAC
GACGAGCACCACCAGGACCTGACCCTGCTGAAAGCTCTCGTGCGGCAGCAGCTGCCTGAGAAGTACAAAG
AGATTTTCTTCGACCAGAGCAAGAACGGCTACGCCGGCTACATTGACGGCGGAGCCAGCCAGGAAGAGTT
CTACAAGTTCATCAAGCCCATCCTGGAAAAGATGGACGGCACCGAGGAACTGCTCGTGAAGCTGAACAGA
GAGGACCTGCTGCGGAAGCAGCGGACCTTCGACAACGGCAGCATCCCCCACCAGATCCACCTGGGAGAGC
TGCACGCCATTCTGCGGCGGCAGGAAGATTTTTACCCATTCCTGAAGGACAACCGGGAAAAGATCGAGAA
GATCCTGACCTTCCGCATCCCCTACTACGTGGGCCCTCTGGCCAGGGGAAACAGCAGATTCGCCTGGATGAC
CAGAAAGAGCGAGGAAACCATCACCCCCTGGAACTTCGAGGAAGTGGTGGACAAGGGCGCTTCCGCCCA
GAGCTTCATCGAGCGGATGACCAACTTCGATAAGAACCTGCCCAACGAGAAGGTGCTGCCCAAGCACAGCC
TGCTGTACGAGTACTTCACCGTGTATAACGAGCTGACCAAAGTGAAATACGTGACCGAGGGAATGAGAAAG
CCCGCCTTCCTGAGCGGCGAGCAGAAAAAGGCCATCGTGGACCTGCTGTTCAAGACCAACCGGAAAGTGA
CCGTGAAGCAGCTGAAAGAGGACTACTTCAAGAAAATCGAGTGCTTCGACTCCGTGGAAATCTCCGGCGTG
GAAGATCGGTTCAACGCCTCCCTGGGCACATACCACGATCTGCTGAAAATTATCAAGGACAAGGACTTCCTG
GACAATGAGGAAAACGAGGACATTCTGGAAGATATCGTGCTGACCCTGACACTGTTTGAGGACAGAGAGAT
GATCGAGGAACGGCTGAAAACCTATGCCCACCTGTTCGACGACAAAGTGATGAAGCAGCTGAAGCGGCGG
AGATACACCGGCTGGGGCAGGCTGAGCCGGAAGCTGATCAACGGCATCCGGGACAAGCAGTCCGGCAAG
ACAATCCTGGATTTCCTGAAGTCCGACGGCTTCGCCAACAGAAACTTCATGCAGCTGATCCACGACGACAGC

CTGACCTTTAAAGAGGACATCCAGAAAGCCCAGGTGTCCGGCCAGGGCGATAGCCTGCACGAGCACATTG
CCAATCTGGCCGGCAGCCCCGCCATTAAGAAGGGCATCCTGCAGACAGTGAAGGTGGTGGACGAGCTCGT
GAAAGTGATGGGCCGGCACAAGCCCGAGAACATCGTGATCGAAATGGCCAGAGAGAACCAGACCACCCA
GAAGGGACAGAAGAACAGCCGCGAGAGAATGAAGCGGATCGAAGAGGGCATCAAAGAGCTGGGCAGCC
AGATCCTGAAAGAACACCCCGTGGAAAACACCCAGCTGCAGAACGAGAAGCTGTACCTGTACTACCTGCAG
AATGGGCGGGATATGTACGTGGACCAGGAACTGGACATCAACCGGCTGTCCGACTACGATGTGGACCATATC
GTGCCTCAGAGCTTTCTGAAGGACGACTCCATCGACAACAAGGTGCTGACCAGAAGCGACAAGAACCGGG
GCAAGAGCGACAACGTGCCCTCCGAAGAGGTCGTGAAGAAGATGAAGAACTACTGGCGGCAGCTGCTGA
ACGCCAAGCTGATTACCCAGAGAAAGTTCGACAATCTGACCAAGGCCGAGAGAGGCGGCCTGAGCGAACT
GGATAAGGCCGGCTTCATCAAGAGACAGCTGGTGGAAACCCGGCAGATCACAAAGCACGTGGCACAGATC
CTGGACTCCCGGATGAACACTAAGTACGACGAGAATGACAAGCTGATCCGGGAAGTGAAAGTGATCACCCT
GAAGTCCAAGCTGGTGTCCGATTTCCGGAAGGATTTCCAGTTTTACAAAGTGCGCGAGATCAACAACTACC
ACCACGCCCACGACGCCTACCTGAACGCCGTCGTGGGAACCGCCCTGATCAAAAAGTACCCTAAGCTGGAA
AGCGAGTTCGTGTACGGCGACTACAAGGTGTACGACGTGCGGAAGATGATCGCCAAGAGCGAGCAGGAAA
TCGGCAAGGCTACCGCCAAGTACTTCTTCTACAGCAACATCATGAACTTTTTCAAGACCGAGATTACCCTGG
CCAACGGCGAGATCCGGAAGCGGCCTCTGATCGAGACAAACGGCGAAACCGGGGAGATCGTGTGGGATA
AGGGCCGGGATTTTGCCACCGTGCGGAAAGTGCTGAGCATGCCCCAAGTGAATATCGTGAAAAAGACCGA
GGTGCAGACAGGCGGCTTCAGCAAAGAGTCTATCCTGCCCAAGAGGAACAGCGATAAGCTGATCGCCAGA
AAGAAGGACTGGGACCCTAAGAAGTACGGCGGCTTCGACAGCCCCACCGTGGCCTATTCTGTGCTGGTGGT
GGCCAAAGTGGAAAAGGGCAAGTCCAAGAAACTGAAGAGTGTGAAAGAGCTGCTGGGGATCACCATCAT
GGAAAGAAGCAGCTTCGAGAAGAATCCCATCGACTTTCTGGAAGCCAAGGGCTACAAAGAAGTGAAAAA
GGACCTGATCATCAAGCTGCCTAAGTACTCCCTGTTCGAGCTGGAAAACGGCCGGAAGAGAATGCTGGCCT
CTGCCGGCGAACTGCAGAAGGGAAACGAACTGGCCCTGCCCTCCAAATATGTGAACTTCCTGTACCTGGCC
AGCCACTATGAGAAGCTGAAGGGCTCCCCCGAGGATAATGAGCAGAAACAGCTGTTTGTGGAACAGCACA
AGCACTACCTGGACGAGATCATCGAGCAGATCAGCGAGTTCTCCAAGAGAGTGATCCTGGCCGACGCTAAT
CTGGACAAAGTGCTGTCCGCCTACAACAAGCACCGGGATAAGCCCATCAGAGAGCAGGCCGAGAATATCAT
CCACCTGTTTACCCTGACCAATCTGGGAGCCCCTGCCGCCTTCAAGTACTTTGACACCACCATCGACCGGAA
GAGGTACACCAGCACCAAAGAGGTGCTGGACGCCACCCTGATCCACCAGAGCATCACCGGCCTGTACGAG
ACACGGATCGACCTGTCTCAGCTGGGAGGCGACAAAAGGCCGGCGGCCACGAAAAAGGCCGGCCAGGCA
AAAAAGAAAAAGTAAGGATCCAGAGACTCTTATCAAGAATCCCATCTCTTGCTTGCTTTTTTTTGTTGCTTCC
CTTTGATAGGGTTTGTTTTTCTTGTTTCAGTGACTTTCTATGTTAAAAGATAATGTCAGTAAAAGGATTTGGTT
TTCTATTATTCTGAATCGATTACGGAAGATTCTTGCTTAATTCCAATCTATACAAGTATCGTGAAATAATGACCG
TTTATGTGGTACCGAGCTCGAATTC

"_":p19-2A, " " : SpCas9-NLS; "ATG": Start codon; "TAA": Stop codon

>p35S-GU-IR-US-polyA

CATGGAGTCAAAGATTCAAATAGAGGACCTAACAGAACTCGCCGTAAAGACTGGCGAACAGTTCATACAGA
GTCTCTTACGACTCAATGACAAGAAGAAAATCTTCGTCAACATGGTGGAGCACGACACACTTGTCTACTCCA
AAAATATCAAAGATACAGTCTCAGAAGACCAAAGGGCAATTGAGACTTTTCAACAAAGGGTAATATCCGGA
AACCTCCTCGGATTCCATTGCCCAGCTATCTGTCACTTTATTGTGAAGATAGTGGAAAAGGAAGGTGGCTCCT
ACAAATGCCATCATTGCGATAAAGGAAAGGCCATCGTTGAAGATGCCTCTGCCGACAGTGGTCCCAAAGAT
GGACCCCCACCCACGAGGAGCATCGTGGAAAAAGAAGACGTTCCAACCACGTCTTCAAAGCAAGTGGATT
GATGTGATATCTCCACTGACGTAAGGGATGACGCACAATCCCACTATCCTTCGCAAGACCCTTCCTCTATATAA
GGAAGTTCATTTCATTTGGAGAGAACACGGGGGACTCTTGACCATGGTAGATCTGAGGGTAAATTTCTAGTT

TTTCTCCTTCATTTTCTTGGTTAGGACCCTTTTCTCTTTTTATTTTTTTGAGCTTTGATCTTTCTTTAAACTGATC
TATTTTTTAATTGATTGGTTATGGTGTAAATATTACATAGCTTTAACTGATAATCTGATTACTTTATTTCGTGTGT
CTATGATGATGATGATAGTTACAGAACCGACGACTCGTCCGTCCTGTAGAAACCCCAACCCGTGAAATCAAA
AAACTCGACGGCCTGTGGGCATTCAGTCTGGATCGCGAAAACTGTGGAATTGATCAGCGTTGGTGGGAAA
GCGCGTTACAAGAAAGCCGGGCAATTGCTGTGCCAGGCAGTTTTAACGATCAGTTCGCCGATGCAGATATTC
GTAATTATGCGGGCAACGTCTGGTATCAGCGCGAAGTCTTTATACCGAAAGGTTGGGCAGGCCAGCGTATCG
TGCTGCGTTTCGATGCGGTCACTCATTACGGCAAAGTGTGGGTCAATAATCAGGAAGTGATGGAGCATCAG
GGCGGCTATACGCCATTTGAAGCCGATGTCACGCCGTATGTTATTGCCGGGAAAAGTGTACGTATCACCGTTT
GTGTGAACAACGAACTGAACTGGCAGACTATCCCGCCGGGAATGGTGATTACCGACGAAAACGGCAAGAA
AAAGCAGTCTTACTTCCATGATTTCTTTAACTATGCCGGAATCCATCGCAGCGTAATGCTCTACACCACGCCGA
ACACCTGGGTGGACGATATCACCGTGGTGACGCATGTCGCGCAAGACTGTAACCACGCGTCTGTTGACTGG
CAGGTGGTGGCCAATGGTGATGTCAGCGTTGAACTGCGTGATGCGGATCAACAGGTGGTTGCAACTGGAC
AAGGCACTAGTTCCCTTTATCTCTTAGGGATA**ACAGGGTAATAGAGATAAA**GGGAGGCCTGAATTGCTGTG
CCAGGCAGTTTTAACGATCAGTTCGCCGATGCAGATATTCGTAATTATGCGGGCAACGTCTGGTATCAGCGC
GAAGTCTTTATACCGAAAGGTTGGGCAGGCCAGCGTATCGTGCTGCGTTTCGATGCGGTCACTCATTACGGC
AAAGTGTGGGTCAATAATCAGGAAGTGATGGAGCATCAGGGCGGCTATACGCCATTTGAAGCCGATGTCAC
GCCGTATGTTATTGCCGGGAAAAGTGTACGTATCACCGTTTGTGTGAACAACGAACTGAACTGGCAGACTAT
CCCGCCGGGAATGGTGATTACCGACGAAAACGGCAAGAAAAAGCAGTCTTACTTCCATGATTTCTTTAACTA
TGCCGGAATCCATCGCAGCGTAATGCTCTACACCACGCCGAACACCTGGGTGGACGATATCACCGTGGTGAC
GCATGTCGCGCAAGACTGTAACCACGCGTCTGTTGACTGGCAGGTGGTGGCCAATGGTGATGTCAGCGTTG
AACTGCGTGATGCGGATCAACAGGTGGTTGCAACTGGACAAGGCACTAGCGGGACTTTGCAAGTGGTGAA
TCCGCACCTCTGGCAACCGGGTGAAGGTTATCTCTATGAACTCGAAGTCACAGCCAAAAGCCAGACAGAGT
CTGATATCTACCCGCTTCGCGTCGGCATCCGGTCAGTGGCAGTGAAGGGCCAACAGTTCCTGATTAACCACA
AACCGTTCTACTTTACTGGCTTTGGTCGTCATGAAGATGCGGACTTACGTGGCAAAGGATTCGATAACGTGC
TGATGGTGCACGACCACGCATTAATGGACTGGATTGGGGCCAACTCCTACCGTACCTCGCATTACCCTTACGC
TGAAGAGATGCTCGACTGGGCAGATGAACATGGCATCGTGGTGATTGATGAAACTGCTGCTGTCGGCTTTC
AGCTGTCTTTAGGCATTGGTTTCGAAGCGGGCAACAAGCCGAAAGAACTGTACAGCGAAGAGGCAGTCAA
CGGGGAAACTCAGCAAGCGCACTTACAGGCGATTAAAGAGCTGATAGCGCGTGACAAAAACCACCCAAGC
GTGGTGATGTGGAGTATTGCCAACGAACCGGATACCCGTCCGCAAGGTGCACGGGAATATTTCGCGCCACT
GGCGGAAGCAACGCGTAAACTCGACCCGACGCGTCCGATCACCTGCGTCAATGTAATGTTCTGCGACGCTC
ACACCGATACCATCAGCGATCTCTTTGATGTGCTGTGCCTGAACCGTTATTACGGATGGTATGTCCAAAGCGG
CGATTTGGAAACGGCAGAGAAGGTACTGGAAAAAGAACTTCTGGCCTGGCAGGAGAAACTGCATCAGCCG
ATTATCATCACCGAATACGGCGTGGATACGTTAGCCGGGCTGCACTCAATGTACACCGACATGTGGAGTGAA
GAGTATCAGTGTGCATGGCTGGATATGTATCACCGCGTCTTTGATCGCGTCAGCGCCGTCGTCGGTGAACAG
GTATGGAATTTCGCCGATTTTGCGACCTCGCAAGGCATATTGCGCGTTGGCGGTAACAAGAAAGGGATCTTC
ACTCGCGACCGCAAACCGAAGTCGGCGGCTTTTCTGCTGCAAAAACGCTGGACTGGCATGAACTTCGGTG
AAAAACCGCAGCAGGGAGGCAAACAAGCTAGCCACCACCACCACCACCACGTGTGAATTACAGGTGACCA
GCTCGAATTTCCCCGATCGTTCAAACATTTGGCAATAAAGTTTCTTAAGATTGAATCCTGTTGCCGGTCTTGC
GATGATTATCATATAATTTCTGTTGAATTACGTTAAGCATGTAATAATTAACATGTAATGCATGACGTTATTTATG
AGATGGGTTTTTATGATTAGAGTCCCGCAATTATACATTTAATACGCGATAGAAAACAAAATATAGCGCGCAA
ACTAGGATAAATTATCGCGCGCGGTGTCATCTATGTTACTAGATCGGG

“_”: U fragment; “....”: G and S fragment; “ “: IR region; “**Bold**”:sgRNA target site; “GGG”:PAM sequences; “ATG“: Start codon; “TAG“: Stop codon
